# Rapid increase in transferrin receptor recycling promotes adhesion during T cell activation

**DOI:** 10.1186/s12915-022-01386-0

**Published:** 2022-08-24

**Authors:** Pascal Rossatti, Gregory M. I. Redpath, Luca Ziegler, Guerric P. B. Samson, Camille D. Clamagirand, Daniel F. Legler, Jérémie Rossy

**Affiliations:** 1 Biotechnology Institute Thurgau (BITg) at the University of Konstanz, CH-8280 Kreuzlingen, Switzerland; 2 EMBL Australia Node in Single Molecule Science, School of Medical Sciences, University of New South Wales, Sydney, Sydney, Australia; 3 Department of Biology, University of Konstanz, Constance, Germany

**Keywords:** T cell activation, Immunological synapse, Iron, Transferrin, Endocytic recycling, Rab5/Rab11, Adhesion, Zap70, Integrins, Flotillin

## Abstract

**Background:**

T cell activation leads to increased expression of the receptor for the iron transporter transferrin (TfR) to provide iron required for the cell differentiation and clonal expansion that takes place during the days after encounter with a cognate antigen. However, T cells mobilise TfR to their surface within minutes after activation, although the reason and mechanism driving this process remain unclear.

**Results:**

Here we show that T cells transiently increase endocytic uptake and recycling of TfR upon activation, thereby boosting their capacity to import iron. We demonstrate that increased TfR recycling is powered by a fast endocytic sorting pathway relying on the membrane proteins flotillins, Rab5- and Rab11a-positive endosomes. Our data further reveal that iron import is required for a non-canonical signalling pathway involving the kinases Zap70 and PAK, which controls adhesion of the integrin LFA-1 and eventually leads to conjugation with antigen-presenting cells.

**Conclusions:**

Altogether, our data suggest that T cells boost their iron importing capacity immediately upon activation to promote adhesion to antigen-presenting cells.

**Supplementary Information:**

The online version contains supplementary material available at 10.1186/s12915-022-01386-0.

## Background

Iron homeostasis represents a key regulator of innate and adaptive immunity and plays a decisive role in inflammatory processes. Many cell types, including macrophages and T cells, import iron to sustain development and effector functions [1, 2]. Iron starvation typically impairs T cell activation, cytokine secretion and proliferation [2–4]. Consequently, reduction of systemic iron levels is a frequent and effective approach to treat T cell-mediated autoimmunity, for instance for patients with multiple sclerosis [5].

The glycoprotein transferrin, which binds two atoms of ferric iron (Fe^3+^), is the main source of bio-available iron. Mammalian cells import iron through transferrin binding to transferrin receptor 1 (CD71, TfR henceforth). After internalisation, iron is reduced to the ferrous form (Fe^2+^) in endosomes, released from transferrin and transported into the cytoplasm, while TfR and transferrin are recycled to the cell surface to recapture ferric iron [6]. Activated T cells strongly upregulate TfR expression within hours [7], to provide for the extra iron needs of T cell proliferation and differentiation. In fact, the absence of TfR prevents the development of pre-T cells in the thymus [8].

T cell activation and formation of the immunological synapse with antigen-presenting cells induces a profound reorganisation of endocytic trafficking [9, 10]. The microtubule organising centre and endocytic machinery relocate to orchestrate polarised trafficking to and from the synapse and regulate surface expression of receptors, adhesion and signalling proteins [11–16]. Accordingly, returning internalised surface molecules back to the plasma membrane is essential to build the immunological synapse and sustain T cell activation. In this context, we have recently uncovered that the T cell receptor (TCR) is recycled by a fast endocytic pathway organised by the membrane protein flotillins, relying on Rab5-positive early endosomes and Rab11a-positive recycling endosomes [16].

Uptake and intracellular trafficking of transferrin and TfR have been extensively investigated, in part because they are a canonical cargo of clathrin-mediated endocytosis (CME). After endocytosis, TfR traffics via Rab5-positive endosomes and is returned to the plasma membrane by Rab11-positive endosomes [6, 17]. Recycling of TfR has also been shown to require the contribution of flotillins, although through a mechanism that remains elusive [18]. Hence, despite the fact that internalisation of TfR and TCR are mediated by CME and a clathrin-independent endocytic pathway respectively [15, 19], their recycling both rely on flotillins, Rab5 and Rab11a.

T cells need iron to assemble the iron-sulphur clusters necessary for the high DNA replication and energy metabolism demands of proliferation and differentiation hours after activation [20]. Yet, it has been shown that they upregulate TfR surface expression within minutes after TCR triggering and that TfR is surprisingly involved in the formation of the immunological synapse [21]. Here, we set out to understand how T cells control transferrin and TfR surface expression upon activation and how this contributes to the formation of the immunological synapse. We show that T cells transiently boost their iron-capturing capacities immediately after TCR triggering, by upregulating at the same time transferrin uptake and TfR surface expression and recycling. We demonstrate that this rapid increase in TfR recycling is supported by the same Rab5-Rab11a flotillin-dependent endocytic axis that sorts TCR for a fast return to the cell surface upon activation. Functionally, we show that iron is required as early as 5 min after TCR triggering for a signalling pathway involving the kinases Zap70 and PAK and leads to increased integrin LFA-1-driven adhesion to ICAM-1 coated surfaces. Accordingly, iron-deprived T cells form less conjugates with antigen-presenting cells. Altogether, our data suggest that TCR triggering is associated with immediate increased iron needs, which are provided for by boosted TfR recycling, and serve to promote formation of the immunological synapse through increased adhesion to antigen-presenting cells.

## Results

### TCR triggering increases TfR endocytosis, surface expression and recycling

T cell activation leads to increased TfR transcription in order to support the iron demand associated with differentiation and clonal expansion following encounter with an antigenic peptide [7, 22–24]. However, resting T cells contain a large intracellular pool of TfR, which suggests the possibility of a fast recruitment to the plasma membrane after TCR triggering. This is typically the case for proteins involved in the first stage of T cell activation such as the kinase Lck [25], the adaptor LAT [26] or the integrin LFA-1 [27]. To investigate this possibility, we designed a set of flow cytometry experiments to quantify how TCR triggering affects endocytic trafficking of transferrin and TfR to and from the cell surface.

**Fig. 1:**
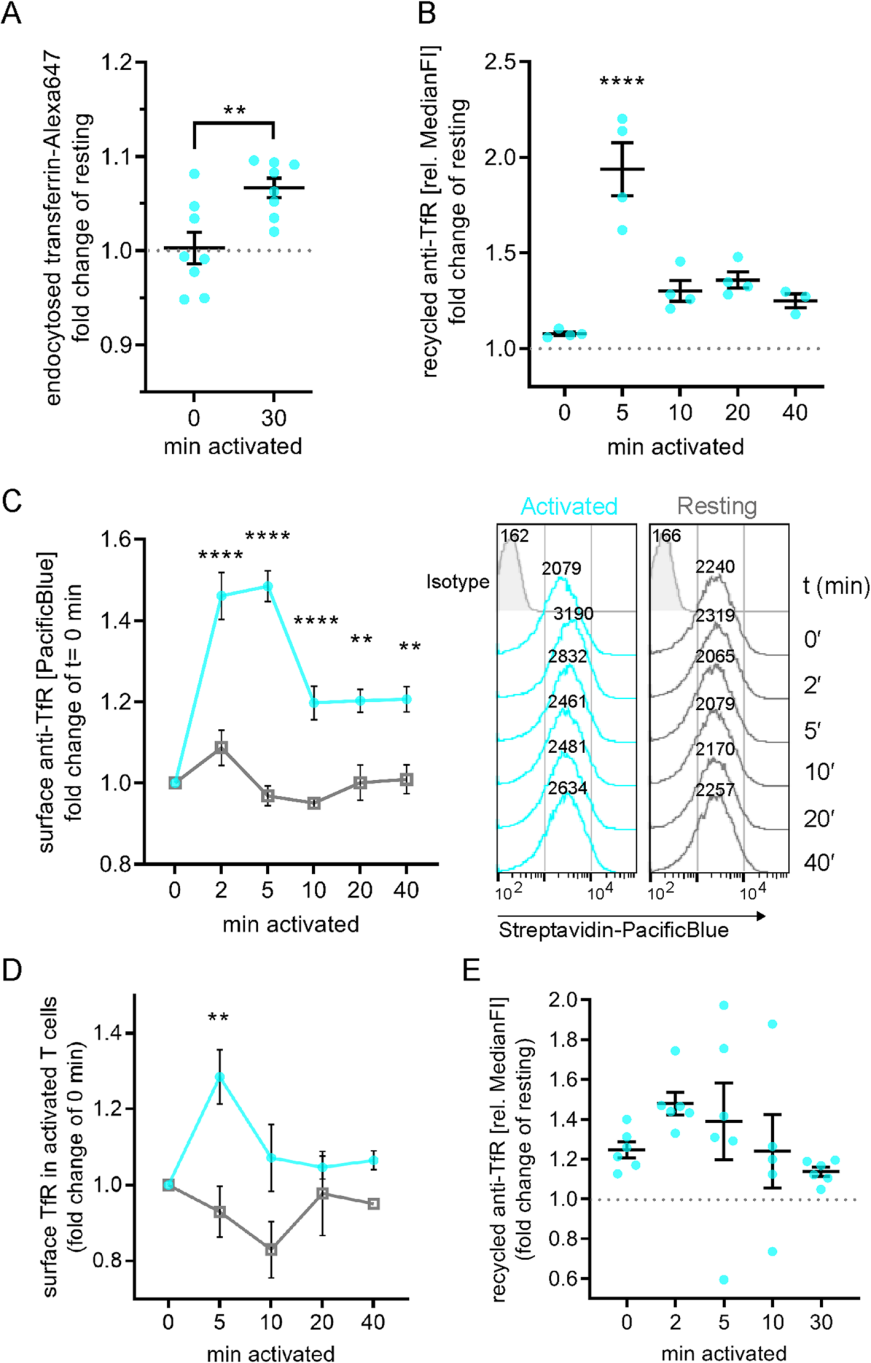
T cell activation stimulates uptake and recycling of transferrin and TfR. **A** Transferrin uptake stimulation upon T cell activation. Jurkat T cells were equilibrated at 37 °C in the presence of transferrin-Alexa647 and subsequently left untouched (resting), or 30 min treated with non-stimulating solution (0 min activated) or 30 min stimulated with activating antibodies anti-CD3ε + anti-CD28. Uptake was stopped on ice and cells were analysed for transferrin-Alexa647 uptake. Displayed is the fold-change of Alexa647 signal relative to untouched (resting) controls. **B** Transiently boosted TfR recycling upon T cell activation. Jurkat T cells were incubated with biotinylated anti-TfR for 90 min to label all TfR pools with antibodies. Then, surface-exposed anti-TfR-biotin was blocked with unlabelled streptavidin. To assess TfR recycling upon activation, cells were activated with soluble activating antibodies (anti-CD3ε + anti-CD28) or left untreated for the indicated times. Antibody-labelled TfR delivered to the surface was detected with Pacific Blue-labelled streptavidin. Fold change of bound streptavidin-Pacific Blue in activated T cells is depicted relative to the corresponding resting cells. **C** Increased surface TfR levels upon T cell activation correlates with boosted TfR recycling. Jurkat T cells were activated with soluble activating antibodies (anti-CD3ε + anti-CD28) or left untreated for the indicated times and immediately fixed with 3.7% paraformaldehyde (PFA). Surface TfR was then stained with biotinylated anti-TfR, which was detected with streptavidin-Pacific Blue. Data points and error bars indicate mean of *n* = 4 independent experiments and standard error of the mean (SEM). **D** Increased surface TfR levels upon activation of expanded primary human T cells. Expanded primary T cells were activated with soluble activating antibodies (anti-CD3ε + anti-CD28) or left untreated for the indicated times and immediately fixed with 3.7% PFA. Surface TfR was then stained with biotinylated anti-TfR, which was detected with streptavidin-Pacific Blue. Data points indicate mean of *n* = 3 independent experiments ± SEM. **E** Increased TfR recycling upon T cell activation. Expanded primary human T cells were treated as described in **B**. Results were analysed and depicted as in **B**. Statistical significance determined with unpaired two-tailed Student’s *t*-test (**A**), one-way ANOVA (**B**, **E**) or two-way ANOVA (**C**, **D**). **p < 0.01; ****p ≤ 0.0001, no indication—not significant

First, we measured uptake of fluorescently labelled transferrin (transferrin-Alexa647) in Jurkat T cells that were unstimulated or activated with antibodies against CD3ε (1.5 μg/ml) and CD28 (1 μg/ml). Activated cells incorporated significantly more transferrin-Alexa647 than resting cells after 30 min (Fig. 1A), illustrating that T cell activation promotes transferrin and TfR endocytosis.

We then measured TfR recycling to determine if the rise in transferrin uptake was correlated with a higher return of the transferrin-TfR complex to the cell surface. We labelled intracellular TfR by feeding Jurkat T cells with a biotinylated antibody against TfR for 90 min and blocked the biotinylated antibodies bound to TfR molecules remaining at the cell surface with unlabelled streptavidin. After incubation with or without activating antibodies against CD3ε and CD28, recycled TfR at the surface was subsequently revealed with labelled streptavidin. TfR recycling was strongly enhanced only 5 min after T cell activation and returned to levels just above baseline from 10 to 40 min of activation (Fig. 1B).

Finally, we determined how increased internalisation and recycling observed upon T cell activation impacted on levels of TfR available for transferrin capture at the cell surface. Surface TfR levels of Jurkat T cells were significantly upregulated between 2 and 40 min after activation compared to resting conditions (Fig. 1C). Of note the highest surface expression levels (149% ± 7%) were reached after 5 min of stimulation, mirroring the time course observed for recycling.

TfR surface levels and endocytic recycling were also increased shortly after TCR and CD28 stimulation in primary human T cells (Fig. 1D, E), confirming our observation in Jurkat T cells. Both recycling and surface expression of TfR peaked at the earliest timepoints measured after introduction of the activating antibodies (2 min and 5 min respectively), as during the measurements performed using Jurkat T cells.

Taken together, our data reveal that T cell activation leads to a fast and transient increase in transferrin and TfR endocytosis, endocytic recycling, and surface expression. These results suggest that T cells boost their capacity to capture and import iron immediately after TCR triggering.

### Increased recycling of TfR is mediated by a fast flotillin-dependent Rab5-Rab11a endocytic axis

**Fig. 2:**
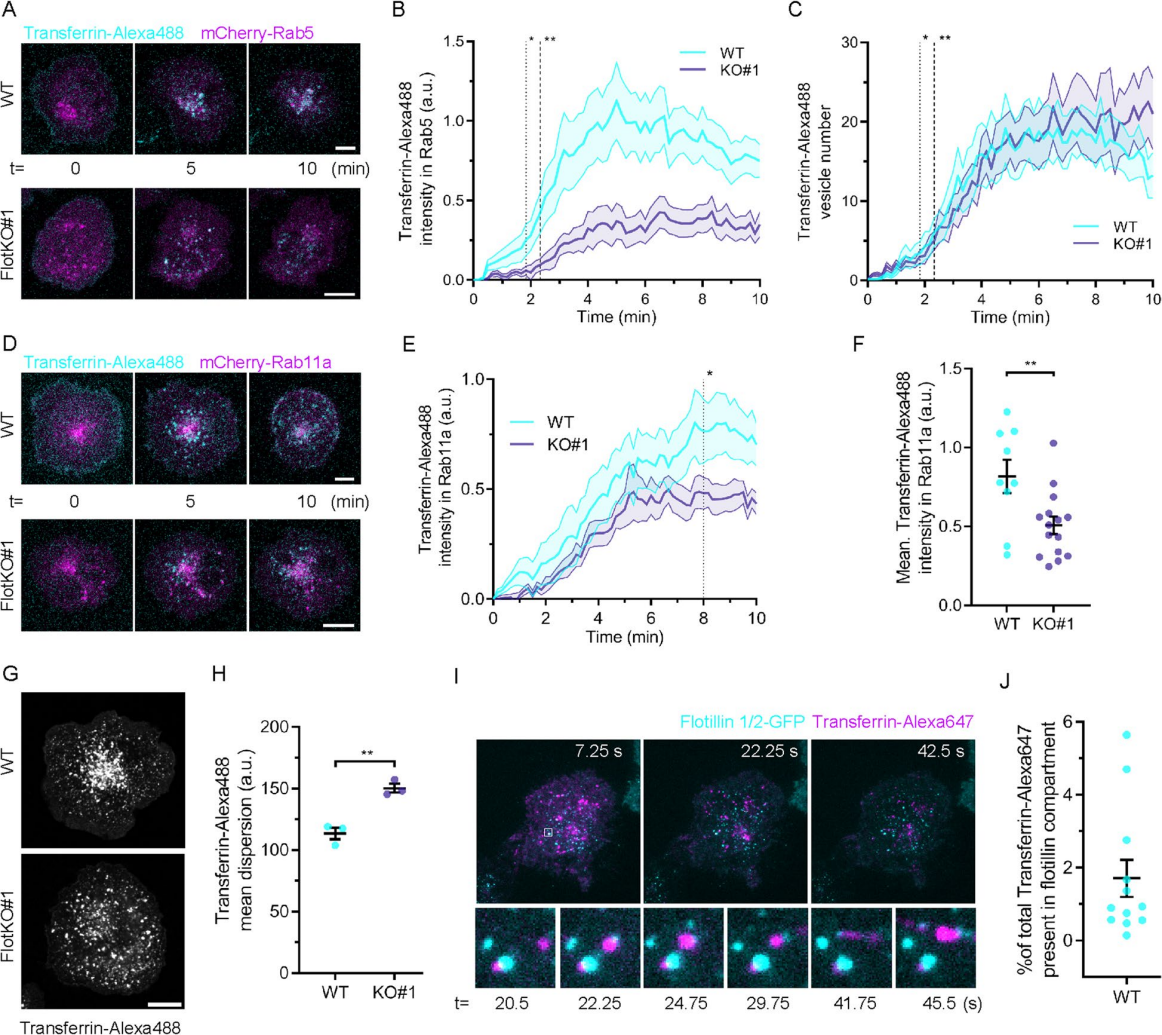
Flotillins mediate transferrin sorting into a Rab5 to Rab11a recycling pathway. **A** Representative confocal images of transferrin-Alexa488 internalisation in WT (left panel) and FlotKO (right panel) Jurkat T cells expressing mCherry-Rab5. **B** Mean transferrin-Alexa488 intensity present in Rab5 endosomes in WT or FlotKO Jurkat T cells over time. Dotted and dashed lines represent the timepoints from which the transferrin-Alexa488 intensity significantly diverges in Rab5 endosomes between WT and FlotKO. Pooled Data from 15 (WT) and 13 (KO#1) imaged cells. **C** Transferrin-Alexa488 vesicle number over time. Dotted and dashed lines represent time of intensity divergence calculated in **B**. Pooled data from 12 (WT) and 10 (KO#1) imaged cells. **D** Representative confocal images of transferrin-Alexa488 internalisation in WT (left panel) and FlotKO (right panel) Jurkat T cells expressing mCherry-Rab11a. **E** Mean transferrin-Alexa488 intensity present in Rab11a endosomes in WT or FlotKO Jurkat T cells over time. Dotted line represents the timepoint from which the transferrin-Alexa488 intensity significantly diverges in Rab11a endosomes between WT and FlotKO. Pooled data from 9 (WT) and 15 (KO#1) imaged cells. **F** Mean transferrin-Alexa488 intensity present in Rab11a endosomes in WT or FlotKO Jurkat T cells during 10 min imaging. **G** Representative confocal images of WT (left) or FlotKO (right) Jurkat T cells incubated with transferrin-Alexa488 for 20 min prior to fixation. **H** Mean fluorescence dispersion of transferrin-Alexa488 in WT and FlotKO Jurkat T cells. **I** Representative TIRF time series of transferrin-positive vesicle transiently interacting with the flotillin-positive compartment. **J** Mean transferrin-Alexa488 intensity present in flotillin-positive compartment in Jurkat T cells during 2-min imaging. Data points indicate single cells (**F, J**) or means of independent experiments (**H**); error bars indicate mean ± SEM. **p < 0.01 from unpaired, two-tailed Student’s *t*-test. Dotted line = *p* < 0.05 and dashed line = *p* < 0.01 from Wilcoxon rank-sum test of pooled data. Scale bars = 5 μm

The rapid time scale of the changes in TfR recycling and surface levels induced by T cell activation suggests that TfR is transported through a fast recycling pathway. Transferrin is a cargo of CME [28] and recycles via Rab11 endosomes [6]. TfR recycling has further been shown to involve membrane organising proteins flotillins [18]. Incidentally, we have recently uncovered a fast endocytic pathway that relies on Rab11a and flotillins that returns internalised TCRζ to the cell surface [16]. Therefore, we sought to determine if the same pathway mediates TfR recycling in activated T cells. We used a live-cell microscopy approach [29] to determine if incorporation of transferrin in Rab5-positive sorting and Rab11a-positive recycling endosomes was impaired in Jurkat T cells where flotillins expression had been knock-out by CRISPR/Cas9 gene editing (FlotKO) [15]. To measure incorporation of transferrin into endosomal compartments positive for Rab5 or Rab11a, cells expressing Rab5- or Rab11a-mCherry were incubated with transferrin-Alexa488 on ice for 5 min, washed, transferred into the microscope at 37 °C and imaged (Fig. 2A, D and Additional File 1: Fig. S1). The transferrin signal present in Rab5 endosomes significantly diverged between WT and FlotKO cells from 50 s following transfer to 37 °C (Fig. 2B, dotted line), when only 3 transferrin vesicles on average had formed (Fig. 2C, dotted line). No difference was observed in the number of vesicles formed over time between WT and FlotKO cells, indicating transferrin endocytosis is not perturbed in the absence of flotillin (Fig. 2C). In agreement with reduced transferrin sorting into Rab5 endosomes, we measured significantly less transferrin sorting into Rab11a endosomes in FlotKO cells compared to WT cells from 8 min after the start of imaging (Fig. 2E, dotted line). Consequently, we detected less transferrin in Rab11a-positive endosomes over 10 min in FlotKO cells compared to WT cells (Fig. 2F).

We have previously shown that the spatial distribution of endosomes constituting the Rab5-Rab11a endocytic axis relies on flotillins in T cells [16]. To further establish the dependence of transferrin sorting and recycling on this pathway, we analysed the spatial distribution of endocytosed transferrin vesicles in Jurkat T cells fixed 20 min after exposure to fluorescently labelled transferrin (Fig. 2G). Similar to what we had observed for Rab5, Rab11a and TCR [16], transferrin-positive vesicles were more dispersed in FlotKO cells (Fig. 2H), further indicating that transferrin sorting to Rab11a-positive endosomes is mediated by the same flotillin-positive endocytic network that sorts TCR for the same intracellular destination. In a previous study, we showed that flotillins promoted incorporation of TCR in Rab5 and Rab11-positive endosomes and regulated their spatial organisation. Nevertheless, flotillin-positive vesicles only moderately co-localised with Rab5 and Rab11 intracellular structures, leading us to the hypothesis that flotillins act on these compartments through transient interactions [16, 30]. Accordingly, even though flotillins were required to sort transferrin into Rab5- and Rab11-positive endosomes and for the spatial organisation of transferrin-positive endosomes, we measured moderate correlation and transient interaction between flotillin and transferrin positive vesicles (Fig. 2I, J).

### Flotillin-deficient T cells fail to increase TfR recycling upon T cell activation

**Fig. 3:**
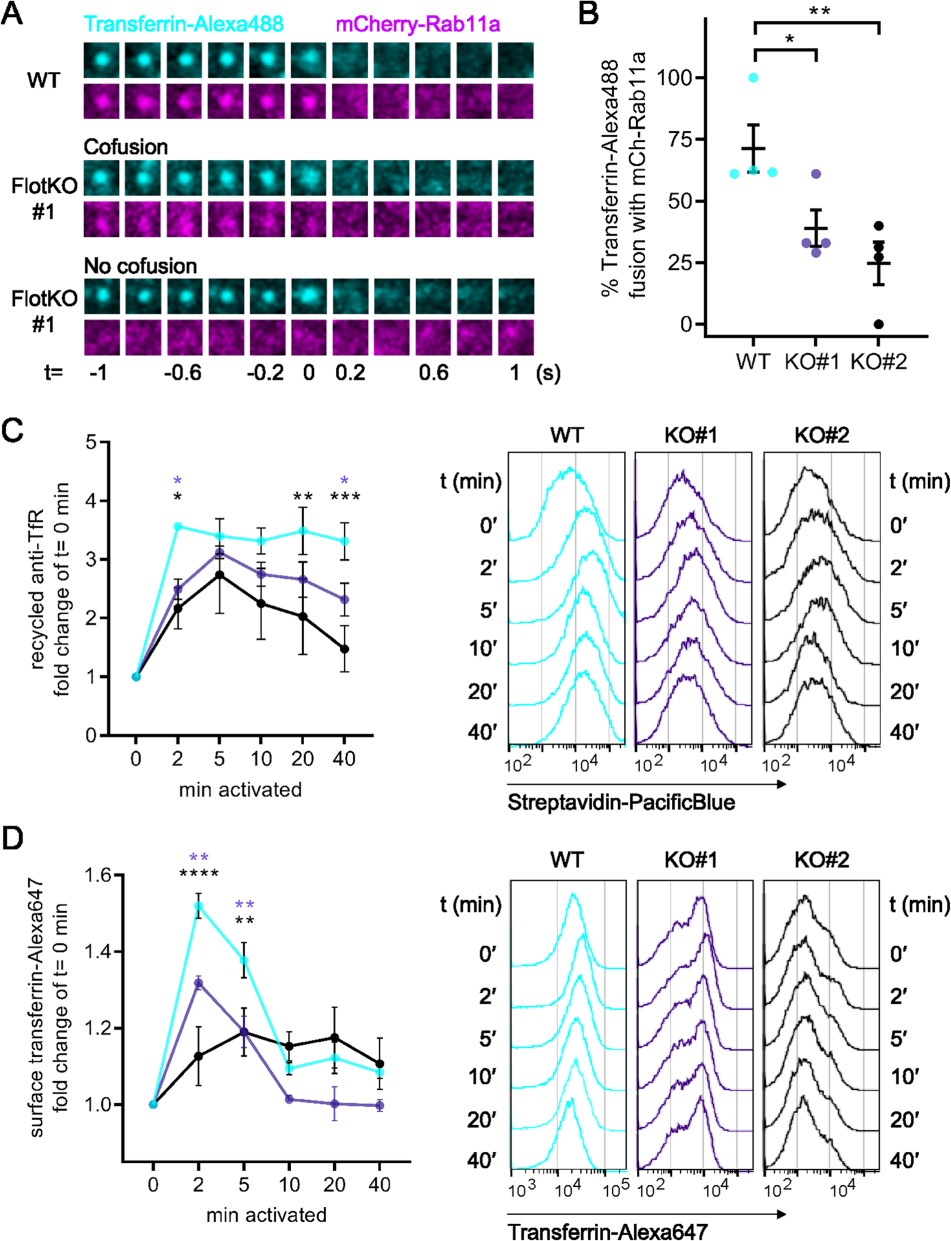
TfR surface delivery and boosted recycling relies on flotillins. **A** Representative TIRF images of transferrin-Alexa488 (cyan) vesicle co-fusion events with mCherry Rab11a (magenta) in WT Jurkat T cells (top) or fusion with (middle) or without (bottom) mCherry-Rab11a in FlotKO Jurkat T cells. **B** Quantification of the percentage of transferrin-Alexa488 fusion events containing mCherry-Rab11a in WT and two FlotKO Jurkat T cell lines. Fusion events were counted as containing the indicated protein if a decrease in maximum intensity from the last three frames was greater than 10% compared to the maximum intensity of the initial frame. Data points represent means of independent experiments. **C** Quantification of TfR recycling boost in WT and FlotKO Jurkat T cells upon activation (left) and representative histograms (right) of *n* = 4 independent experiments. Cells were incubated with biotinylated anti-TfR for 90 min to label all TfR pools with antibodies. Then, surface-exposed anti-TfR-biotin was blocked with unlabelled streptavidin. To assess TfR recycling, cells were activated with soluble antibodies (anti-CD3ε + anti-CD28) or left untreated for the indicated times. Antibody-labelled TfR delivered to the surface was detected with Pacific Blue-labelled streptavidin. Fold change of bound streptavidin-Pacific Blue in activated T cells is depicted relative to resting cells at timepoint 0 min. **D** Increased surface TfR levels upon T cell activation in WT vs FlotKO Jurkat T cells. Quantification (left) and representative histograms (right) of *n* = 3 independent experiments. Cells were activated with soluble antibodies (anti-CD3ε + anti-CD28) or left untreated for the indicated times. Surface TfR was then stained with transferrin-Alexa647. Statistical significance determined with one-way ANOVA (**B**) or two-way ANOVA (**C**, **D**).*p ≤ 0.05; **p < 0.01; ***p < 0.001; ****p < 0.0001, no indication—not significant

Our data show that flotillins are required to sort transferrin into Rab11a-positive recycling compartments after endocytosis. The final step of endocytic recycling is the return of internalised surface molecules by Rab11a-positive vesicles for fusion with the plasma membrane [30]. Thus, we used two-colour total internal reflection fluorescence (TIRF) microscopy to visualise and quantify the delivery of endocytosed transferrin to the plasma membrane by Rab11a-positive vesicles in WT and FlotKO Jurkat T cells. We defined two categories of fusion events of transferrin with the plasma membrane: fusion with or without Rab11a (Fig. 3A). Transferrin co-fusion with Rab11a was significantly lower in both FlotKO cell lines compared to WT cells (42.3 ± 9.3% KO#1, 22.4 ± 11.8% KO#2 vs. 74.5 ± 12.8 for WT, Fig. 3B), confirming that transferrin sorting into Rab11a transport vesicles is impaired in FlotKO cells.

Next, we used the same flow cytometry approach described in Fig. 1 to determine how impaired delivery of transferrin to the Rab11a recycling apparatus impacted the recycling and surface expression of the transferrin-TfR complex. TfR recycling was reduced in FlotKO cells as early as 2 min after TCR activation (Fig. 3C), consistent with the role of flotillins in sorting internalised transferrin and TfR to Rab11a-positive recycling endosomes. The total surface levels of transferrin were also lower in FlotKO compared to WT Jurkat T cells within the first 10 min of activation (Fig. 3D). Crucially, these results further confirm that flotillin-dependent endocytic sorting is required for the sharp and transient increase of TfR surface expression that we observed between 0 and 5 min after TCR triggering. Altogether, these data demonstrate that without flotillins, the transferrin-TfR complex is less efficiently sorted into Rab11a recycling endosomes and consequently fails to be enriched at the cell surface within the first 5 min following TCR triggering.

### Phosphatidylserine demarks fast endocytic recycling pathway of transferrin-TfR

We next sought to characterise the fast recycling of transferrin further, in terms of lipid content of the endosomes that constitute this pathway. The lipid component phosphatidylserine (PS) represents one major regulatory factor for the identity of the membrane domains that organise specific endocytic trafficking routes [30]. Coming from the inner leaflet of the plasma membrane, PS remains detectable within endosomes taking the Rab11a recycling route via Rab5 early endosomes [31, 32]. Importantly, PS-positive endosomes have often been shown to contain TfR [31–35].

**Fig. 4:**
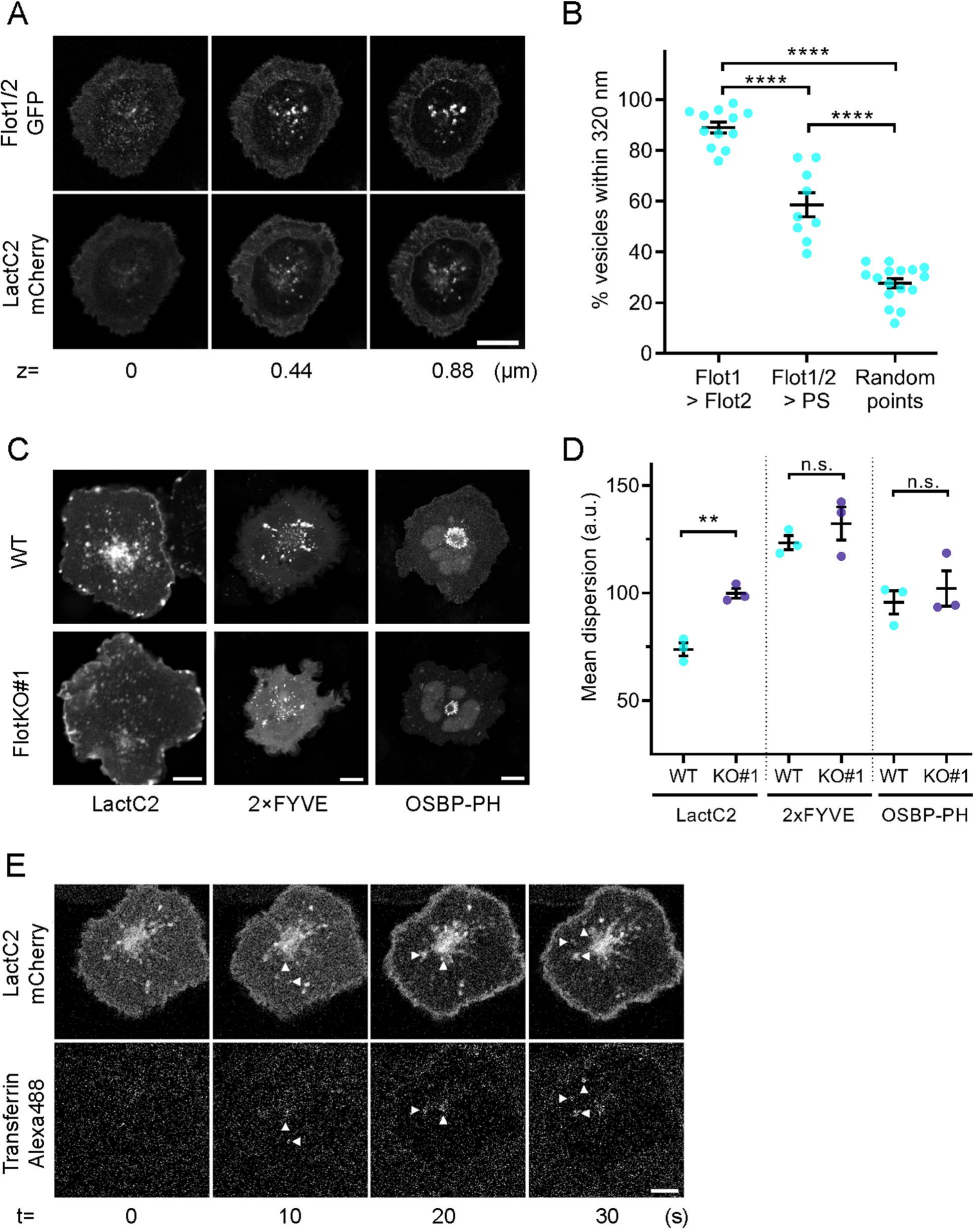
Fast transferrin-TfR recycling axis is demarked by phosphatidylserine. **A** Representative confocal images at 3 different z-levels at (0 μm) and above (0.44 μm and 0.88 μm) the immunological synapse of activated Jurkat T cells expressing Flot1/2-GFP and the PS-sensor LactC2-mCherry. **B** Nearest neighbour analysis between green and red channel identifying % of vesicles positive for both colours (≤ 320 nm apart; % Flot1 vesicles also containing Flot2, % Flot1/2 vesicles positive for LactC2 (PS) and random points to each other) in GFP and mCherry as described in [15]. **C** Representative confocal images of fixed, 20-min activated WT (upper) or FlotKO (lower) Jurkat T cells expressing PS sensor LactC2-GFP, PI(3)P-sensor 2 × FYVE-GFP or PI(4)P-sensor OSBP-PH-GFP. Scale bar = 5 μm. **D** Mean fluorescence dispersion of GFP channel in WT and FlotKO Jurkat T cells. **E** Representative time series of confocal imaging of an activated Jurkat T cell expressing the PS-sensor Lact-C2-mCherry during addition of transferrin-Alexa488. Error bars indicate mean ± SEM. Statistical significance determined with one-way ANOVA (**B)** or unpaired two-tailed Student’s *t*-test (**D**). Data points represent single live-imaged cells (**B**) or means of independent experiments (**D**). **p < 0.01; ****p ≤ 0.0001, n.s.—not significant

We first investigated the correlation in time and space between vesicles positive for flotillins and PS, visualised by Lact-C2-mCherry, using a cross-channel nearest-neighbour analysis [15]. As flotillin-1 and flotillin-2 act as obligate heterodimers/tetramers [36], we co-transfected GFP-fused flotillin-1 and untagged flotillin-2 (referred to as Flot1/2-GFP hereafter) to ensure both were present in equal quantities to form functional flotillin microdomains. We found a high percentage of endosomes that were positive for both, Flot1/2-GFP and phosphatidylserine (58% ± 14%, Fig. 4A, B), indicating that flotillins likely regulate the organisation of the PS-positive endosomal network. Importantly, we observed that PS-positive endosomes were significantly more dispersed in activated FlotKO cells compared to WT cells (Fig. 4C, D), indicating that like transferrin containing endosomes, their spatial organisation relies on flotillins. In contrast, the spatial distribution of PI(3)P-positive endosomes (predominantly early endosomes)—visualised with the sensor 2xFYVE-mCherry—and PI(4)P-positive endomembranes (predominantly Golgi-related compartments)—visualised with the mCherry-tagged PH domain of OSBP—were unaffected by the absence of flotillins, highlighting the specificity of the membrane scaffolding properties of flotillin-1 and flotillin-2 for defined subsets of endocytic compartments.

In agreement with the fact that both PS and transferrin-positive endosomes are linked to flotillins, we found that endosomes involved in transferrin trafficking in T cells also contain PS. From the moment they were formed, transferrin-containing vesicles were positive for PS, visualised by LactC2-mCherry (Fig. 4E). Notably, internalised TCRζ was also found in endosomes positive for PS (Additional File 1: Figs. S2A, B). These data strengthen the hypothesis that despite the fact they are internalised through different pathways [19, 28], TfR and TCR are returned to the surface of activated T cells through a common fast Rab5-Rab11a recycling axis constituted of endosomes positive for PS.

### Blocking transferrin uptake selectively impairs phosphorylation of the kinase Zap70

We showed that T cells route the transferrin-TfR complex through a fast endocytic recycling pathway to sustain a transient increase in transferrin surface expression immediately after TCR stimulation. However, the functional importance of this to T cell activation remains elusive. We hypothesised that increased surface expression of TfR translates into enhanced uptake of iron.

**Fig. 5:**
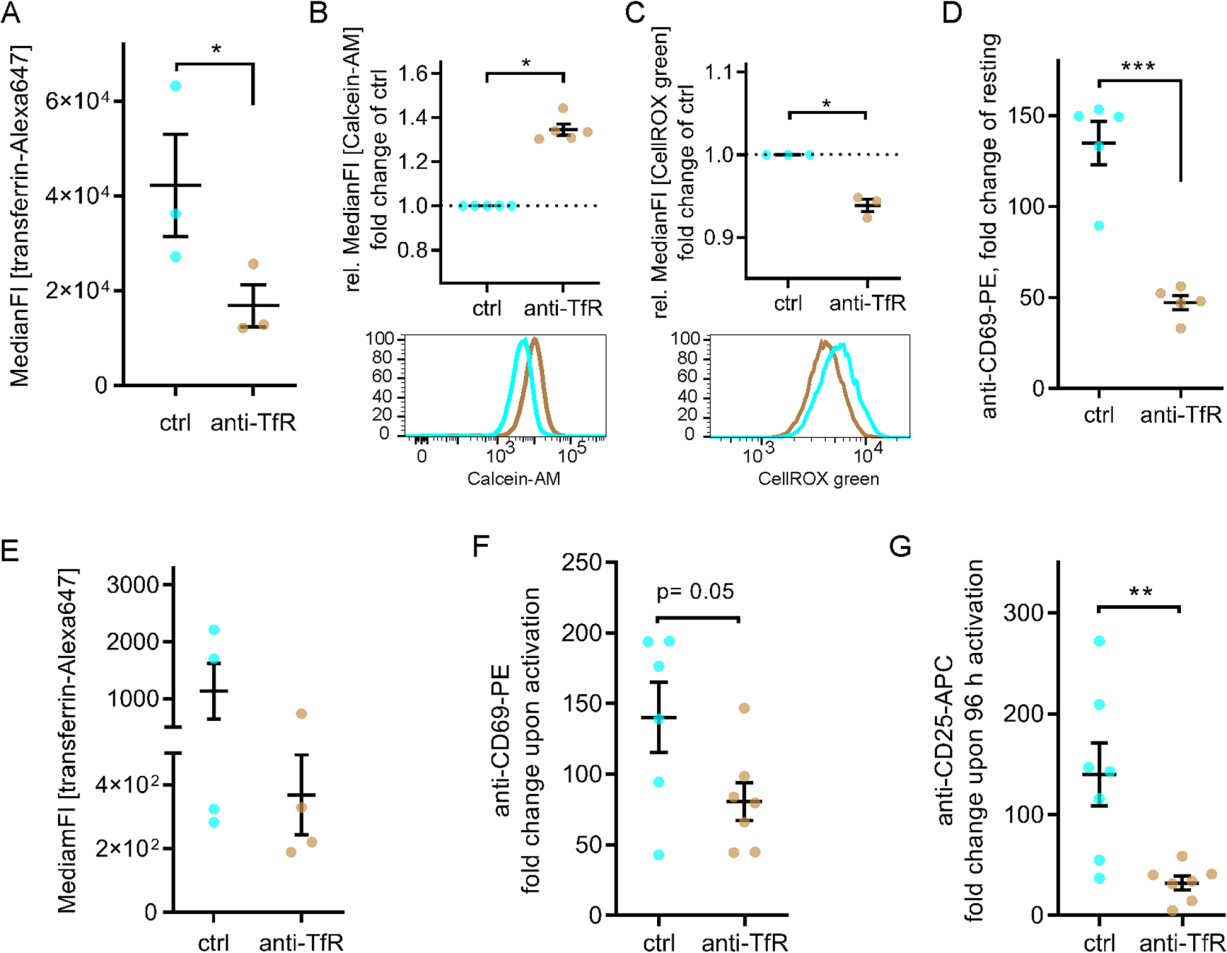
T cell activation requires iron supply via transferrin-TfR system. **A** Effectivity of transferrin uptake blockade with anti-TfR (10 μg/ml) in activated Jurkat T cells. Cells were pre-treated with anti-TfR or not, subsequently incubated at 37 °C for 90 min with 20 μg/ml transferrin-Alexa647 and analysed using flow cytometry. **B** Levels of intracellular labile iron pool (LIP) in anti-TfR-treated versus untreated, activated Jurkat T cells as determined by staining with the iron sensor dye Calcein-AM. Lower fluorescence intensity corresponds to higher LIP levels. **C** Levels of intracellular reactive oxygen species (ROS) in anti-TfR-treated versus untreated, 5-min activated Jurkat T cells as determined by staining with the ROS sensor dye CellROX green. **D** Upregulation of CD69 upon activation on plate-bound anti-CD3ε + anti-CD28. Jurkat T cells were pre-treated or not with anti-TfR and subsequently incubated for 20 h in coated wells for activation. Anti-TfR treatment was maintained throughout the whole experiment where applicable. Surface CD69 expression was determined by staining with anti-CD69-PE and subsequent flow cytometry. **E** Effectivity of transferrin uptake blockade with anti-TfR (10 μg/ml) in 20-h activated primary T cells (activated on plate bound anti-CD3ε + anti-CD28). Cells were pre-treated with anti-TfR or not, subsequently incubated at 37 °C for 90 min with 30 μg/ml transferrin-Alexa647 and analysed using flow cytometry. **F** Upregulation of CD69 upon activation on plate-bound anti-CD3ε + anti-CD28. Primary T cells were pre-treated or not with anti-TfR and subsequently incubated for 20 h in coated wells for activation. Anti-TfR treatment was maintained throughout the whole experiment where applicable. Surface CD69 expression was determined by staining with anti-CD69-PE and subsequent flow cytometry. **G** Upregulation of CD25 upon activation on plate-bound anti-CD3ε + anti-CD28. Primary T cells were pre-treated or not with anti-TfR and subsequently incubated for 96 h in coated wells for activation. Anti-TfR treatment was maintained throughout the whole experiment where applicable. Surface CD25 expression was determined by staining with anti-CD25-APC and subsequent flow cytometry. Data points represent individual experiments. Statistical significance determined with unpaired two-tailed Student’s *t*-test (**A**, **D–G**) or one-sample t-test (**B**, **C)**. *p < 0.05; **p ≤ 0.01; ***p ≤ 0.001; no indication—not significant

To test this hypothesis, we could not use the flotillin KO Jurkat T cells, as we have shown previously flotillin-dependent endocytic trafficking was required for recycling of TCR [15, 16]. Consequently, activation of flotillin KO Jurkat T cells was impaired, showing reduced signalling, nuclear translocation of transcription factors or surface expression of activation markers. Therefore, to specifically investigate the effects of reduced iron uptake without more global effects on T cell that would result from the flotillin knock-out, we used a monoclonal anti-TfR antibody to specifically block transferrin-mediated iron-uptake in Jurkat T cells. We first confirmed that treatment with 10 μg/ml anti-TfR mAb successfully reduced the uptake of fluorescently labelled transferrin in activated Jurkat T cells (Fig. 5A). We then verified the effectiveness of transferrin uptake blockade by measuring the intracellular labile iron pool (LIP) with Calcein upon activation (Fig. 5B). We measured an increased median fluorescence intensity (35% ± 5.6%) in cells treated with anti-TfR compared to control cells, which corresponds to a significant reduction of LIP levels, as binding of free iron to Calcein quenches its fluorescence. As previously reported [37], decrease of LIP in anti-TfR treated cells translated into reduced production of reactive oxygen species (ROS), as quantified by staining with the sensor dye CellROX green (Fig. 5C).

To verify that decreased iron supply affects Jurkat T cell activation, we assessed the expression of the early activation marker CD69 in response to activation by immobilised antibodies against CD3ε and CD28. Upregulation of CD69 was significantly impaired in anti-TfR-treated Jurkat T cells compared to untreated controls after 20 h (Fig. 5D), in agreement with previous studies showing iron-dependence of T cell activation [2, 4].

We next confirmed that blocking transferrin uptake with anti-TfR also impaired primary T cell activation. Anti-TfR treatment greatly reduced uptake of transferrin-Alexa647 by primary T cells (Fig. 5E) and like in Jurkat T cells, impaired in vitro activation as indicated by reduced CD69 and CD25 upregulation (Fig. 5F, G).

**Fig. 6:**
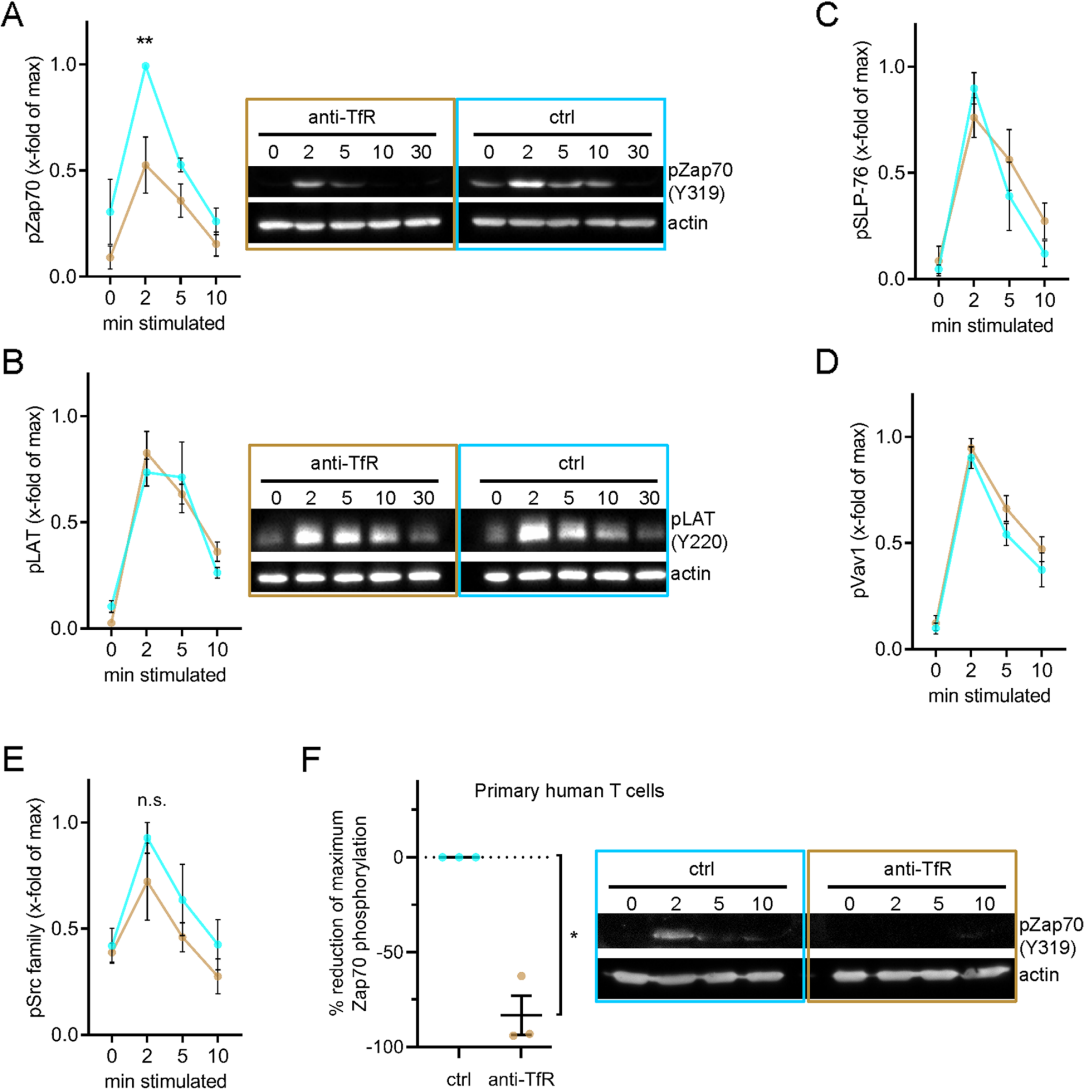
Phosphorylation of Zap70 selectively requires iron supply via transferrin-TfR system. **A**–**E** Immunoblots with phosphorylation-specific antibodies against proteins involved in proximal TCR signalling. Jurkat T cell activation was accomplished with soluble anti-CD3ε + anti-CD28 for the indicated times before cell lysis. Data from 4–5 independent experiments. **A** After blotting, the nitrocellulose membrane was probed with anti-phospho-Zap70 (Y319). Beta actin was used as loading control. Left: Band intensity quantification. Right: Relevant parts of one representative immunoblot. **B** Membrane was probed with anti-phospho-LAT (Y220). After membrane stripping, beta actin was used as loading control. Left: Quantification of band intensities. Right: Relevant parts of one representative immunoblot. **C** After blotting, the nitrocellulose membrane was probed with anti-phospho-SLP-76 (Y145). Beta actin was used as loading control. **D** Membrane was probed with anti-phospho-Vav1 (Y174). Beta actin was used as loading control. **E** After blotting, the nitrocellulose membrane was probed with anti-phospho-Src family (Y416). Beta actin was used as loading control. **F** Primary T cell activation was accomplished with soluble anti-CD3ε + anti-CD28 for the indicated times before cell lysis and SDS PAGE. After blotting, the nitrocellulose membrane was probed with anti-phospho-Zap70 (Y319). Beta actin was used as loading control. Left: Quantification of reduction in Zap70 phosphorylation. Right: Relevant parts of one representative immunoblot. Error bars indicate mean ± SEM. Statistical significance determined with two-way ANOVA (**A–E**) or one-sample t-test (**F)**. *p < 0.05; **p ≤ 0.01; no indication—not significant

Our data revealed that transferrin and TfR surface expression and recycling are upregulated within less than 2 min, peak at 5 min and return to levels just above the baseline after 10 min (Fig. 1). This time course closely matches key signalling events that take place shortly after TCR triggering such as the phosphorylation of the kinase Zap70 [38, 39], the adaptor proteins LAT [38, 39] and SLP-76 [39] or the guanine exchange factor Vav1 [40, 41]. Thus, we performed Western blotting using phospho-specific antibodies against these signalling proteins on lysate of cells stimulated with anti-CD3ε and anti-CD28 and treated or not with anti-TfR. While blocking transferrin uptake had no effect on the phosphorylation of LAT, SLP-76, and Vav1, it significantly reduced phosphorylation of Zap70 at 2 min (Fig. 6A–D). Phosphorylation of the active site of the kinases Lck and Fyn, which are upstream of Zap70 in the TCR signalling pathway, showed a tendency to a slight reduction upon blockade of transferrin uptake, although the difference with control cells was not significant (Fig. 6E). Zap70 phosphorylation was also impaired in activated primary human T cells treated with anti-TfR (Fig. 6F and Additional File 1: Fig. S3A).

### Iron deprivation reduces PAK-dependent integrin adhesion and conjugate formation

**Fig. 7:**
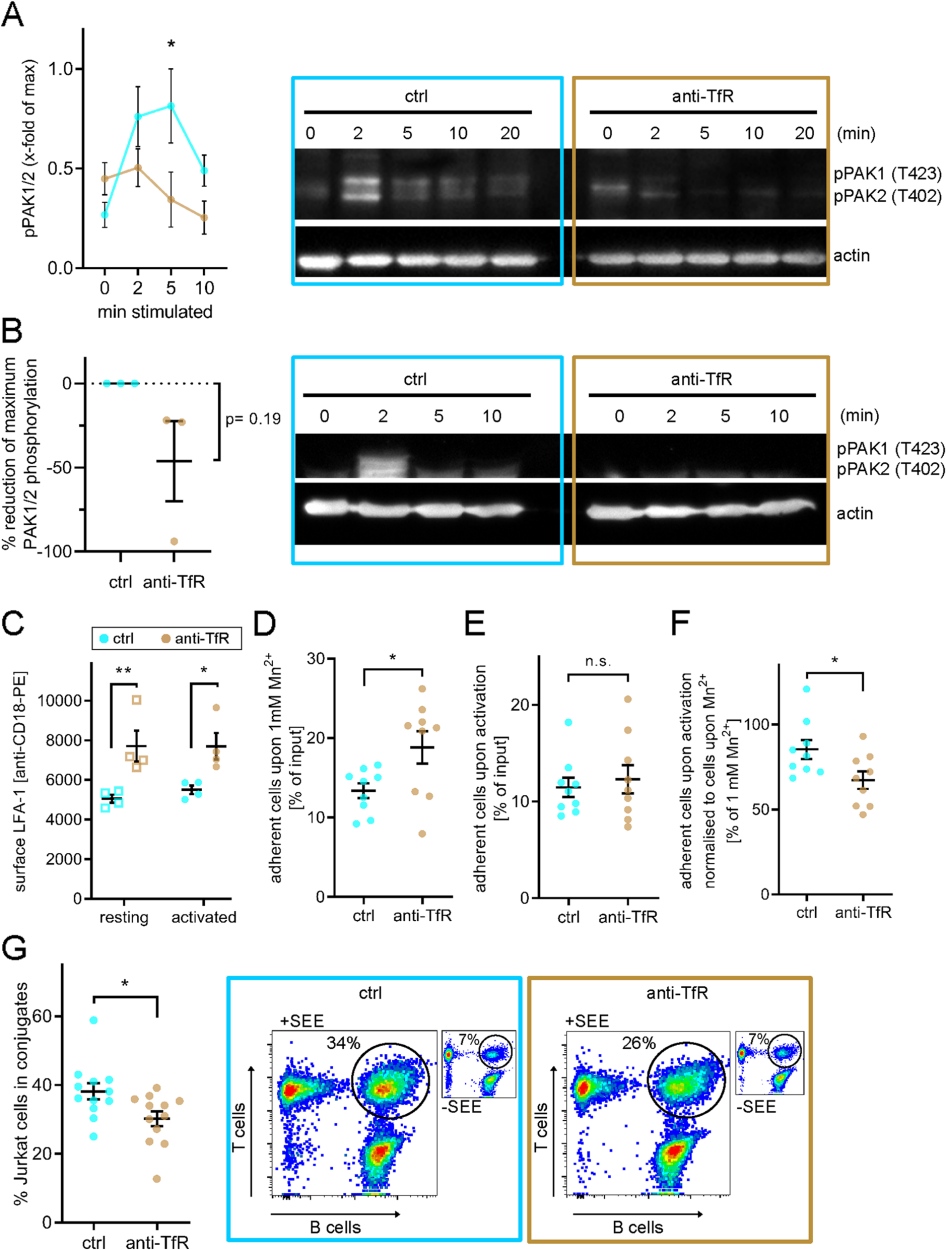
Blockade of iron-uptake via transferrin-TfR axis negatively affects T cell adhesion via integrins and consequently T cell: APC conjugate formation. **A**, **B** Immunoblot with a phosphorylation-specific antibody against PAK1/2 (T423 and T402, respectively). **A** Jurkat T cell activation was accomplished with soluble anti-CD3ε + anti-CD28 for the indicated times before cell lysis. Data from 3 independent experiments. Left: Band intensity quantification. Right: Relevant parts of one representative immunoblot. **B** Expanded primary T cells were treated with anti-TfR or left untreated. Activating antibodies (anti-CD3ε + anti-CD28) were added for the indicated times before lysis. PAK1/2 phosphorylation was detected in T cells of a fraction of donors. An exemplary immunoblot from “responder” T cells is shown. **C** Levels of surface LFA-1 measured using an antibody against the beta-subunit CD18 (ITGb2) on Jurkat T cells treated as indicated. **D** Maximum adhesive potential via LFA-1 upon 30-min treatment with 1 mM MnCl_2_ forcing every LFA-1 surface molecule into high affinity conformation. **E** Adhesion of 30-min activated control and anti-TfR treated Jurkat T cells on ICAM-1 coating. **F** Adherent anti-TfR or untreated Jurkat T cells on ICAM-I coating upon 30-min activation with soluble anti-CD3ε and anti-CD28 relative to the maximum adhesion capacity induced by addition of 1 mM MnCl_2_. **G** Quantification of specific activation-induced conjugate formation between anti-TfR treated or untreated Jurkat T cells and SEE-pulsed Raji B cells. Jurkat T and Raji B cells were stained with CTV and CFSE, respectively, with dye switching between experiments. Cells were mixed in a 1:1 ratio and conjugates formed for 15 min at 37 °C in a pellet. Data displayed as % of Jurkat T cells that formed conjugates with Raji B cells. Unspecific conjugates (Jurkat with un-pulsed Raji B cells) were subtracted for each corresponding sample. Error bars indicate mean ± SEM. Statistical significance determined with two-way ANOVA (**A**, **C**), or one-sample *t*-test (**B**) or with unpaired two-tailed Student’s *t*-test (**D–G**). *p < 0.05; ***p ≤ 0.001; n.s—not significant

The specific effect of transferrin uptake blockade on Zap70 was unexpected, as phosphorylation of LAT, SLP-76 and Vav1 is linked to Zap70 in the classic signalling pathway downstream of TCR [42]. However, it has been shown that Zap70 phosphorylation can be disconnected from the canonical TCR signalling cascade [43] and act in the context of integrin-mediated adhesion, independent of its kinase activity [44]. In this scenario, Zap70 specifically activates p21-activated kinases (PAK1) [45]. This pathway operates in parallel to the SLP-76-dependent activation of PAK1 and relies on the recruitment of GIT and PIX to the immunological synapse [46, 47]. In line with this, we found that phosphorylation of PAK1/2 was impaired at 2 min following TCR stimulation in Jurkat T cells treated with anti-TfR (Fig. 7A). Phosphorylation of PAK1/2 upon TCR and CD28 stimulation was only detected in primary T cells from a fraction of donors (50%, 3/6). Nevertheless, PAK1/2 phosphorylation was impaired upon treatment with anti-TfR in these “responder” T cells (Fig. 7B and Additional File 1: Fig. S3B).

Next, we tested if the effect of iron deprivation on the Zap70-PAK1/2 pathway translated in impaired adhesion capabilities of activated T cells. We chose to focus on binding of the integrin LFA-1 (CD11a/CD18) to the adhesion protein ICAM-1, as this adhesive interaction is essential for formation of the immunological synapse [48, 49]. Surprisingly, when treated with anti-TfR, Jurkat T cells expressed more LFA-1 on the cell surface (Fig. 7C). We next assessed adhesion to ICAM-1-coated surfaces. In this assay, we used MnCl_2_, which switches integrins into a high affinity binding state [50]. Resting Jurkat T cells treated with anti-TfR adhered more to ICAM-1-coated surfaces following MnCl_2_ exposure than untreated cells, as expected considering their increased LFA-1 expression (Fig. 7D). However, upon TCR activation with anti-CD3ε and treatment with anti-TfR, Jurkat T cells did not adhere more to ICAM-1 than cells activated with anti-CD3ε only, despite the fact they had more available LFA-1 (Fig. 7E). Failure of iron-deprived T cells to convert higher LFA-1 surface expression into higher adhesion to ICAM-1 in response to TCR stimulation became apparent after normalisation to the highest possible adhesion induced by MnCl_2_ (Fig. 7F). In summary, these results indicate that iron uptake is required to promote proper cell adhesion to ICAM-1 in response to T cell activation.

Interestingly, staining of activated cells with a specific antibody did not reveal that treatment with anti-TfR prevents induction of the LFA-1 high affinity conformation (Additional File 1: Fig. S4A). Instead, it appears that impaired activation-induced adhesion of anti-TfR-treated cells to ICAM-1 was caused by their inability to mobilise LFA-1 upon TCR triggering. While we measured a moderate but consistent increase of LFA-1 surface expression (9.2%) upon activation of Jurkat T cells, this was not the case in cells that had been pre-treated with antibodies against TfR (0.1%, Fig. 7C). Of note, the interaction of the integrin VLA-4 (CD49d/CD29) with its ligand VCAM-1, which is not present at the IS, was not impaired in anti-TfR-treated Jurkat T cells (Additional File 1: Fig. S4B), highlighting the specificity of the effects of iron blockade on immune synapse-related integrins.

Finally, we verified consequences of impaired adhesion of LFA-1 to ICAM-1 on the formation of the immunological synapse under restricted iron import conditions. To do so, we measured the number of conjugates formed between differentially stained Jurkat T cells and Raji B cells, which had been pulsed with super-antigen (SEE). According to the results of the adhesion assays, we detected less Jurkat-Raji conjugates after treatment of the Jurkat cells with anti-TfR (Fig. 7G), confirming the importance of iron capture for the formation of a stable immunological synapse.

## Discussion

T cell stimulation readily increases surface expression of transferrin receptor [21], in a similar way it does for proteins that are essential for T cell activation or formation of the immunological synapse such as Lck, LAT or LFA [25–27]. However, the mechanism involved and the reason for mobilising an iron importing receptor so early during the process of T cell activation remain unclear. In this study, we show that the enhanced surface expression of TfR is concurrent with increased endocytic recycling within the first 5 min following TCR triggering. Hence, our data show that T cells boost their capacity to import iron immediately after activation, indicating that TfR is likely essential also for T cell activation. We further show that they do so by directing internalised TfR to Rab11a-positive recycling endosomes through a fast endocytic sorting pathway relying on the membrane proteins flotillins, which we had previously identified for TCR [16].

Classically, rapid recycling of surface receptors, including TfR, has been associated with Rab4-positive vesicles [51], by contrast to Rab11a-positive slow recycling compartments [6]. The current understanding is that Rab4-positive recycling vesicles bud directly from Rab5-positive early endosomes and are re-delivered directly to the plasma membrane, while receptors taking the Rab11a route need to be transported all the way to perinuclear compartments before being returned to the cell surface [52]. However, what has been characterised in larger HeLa cells or fibroblast cells might not apply to lymphocytes that have a very confined cytoplasmic volume. In activating T cells, the entire endocytic machinery, including Rab5, Rab4 and Rab11a compartments, is compacted between the nucleus and the immunological synapse [16]. Therefore, is it likely that in these cells faster recycling is not necessarily achieved by shortening distances travelled by endocytic vesicles, but rather by routing cargos to recycling compartments as soon as possible after endocytosis.

Such pre-early endosome sorting steps have been described in other cells [30] and typically involve microtubule-based molecular motors [53]. In this context, flotillins interact with ninein, a dynein motor adaptor [54], and have been reported to promote anchoring of transport vesicles to dynein [55]. Our results are consistent with a flotillin-dynein connection, as they show that flotillins are required for centripetal accumulation of vesicles positive for transferrin and PS, as well as Rab5, Rab11a and TCR in a previous report [16]. Hence, our data strongly suggest that flotillins orchestrate pre-early endosome sorting of surface receptors internalised trough clathrin-mediated (TfR) and trough clathrin-independent routes (TCR) by anchoring a specific population of PS-positive vesicles to dynein.

PS is enriched at the plasma membrane and throughout the endosomal recycling system [56]. Thus, the role of PS could possibly be to provide a membrane environment required for recruitment of recycling regulatory proteins, analogous to the role of phosphoinositides throughout endosomal compartments [30]. Supporting this hypothesis, PS maintains flotillin localisation at the plasma membrane [57], is required for localisation of the scission protein EHD1 on recycling endosome tubules [58] and localises Rab11FIP proteins to recycling endosomes [59]. It has been further shown that depletion of ATP8A1, which maintains the required cytoplasmic orientation of PS on recycling endosomes, disrupts EHD1 recruitment and inhibits transferrin redelivery to the plasma membrane [58]. Hence, we hypothesise that the connection between flotillins and PS could facilitate sorting of cargoes into recycling endosomes immediately following endocytosis, although the protein complement promoting flotillin-mediated sorting into phosphatidylserine-positive endosomes remains to be identified.

One distinctive feature of the upregulation of TfR recycling and surface expression that we observed upon TCR triggering is the fact that they peak at 5 min and return to values just above baseline after 10 min. However, a closer look at the literature reveals that this is not unique to TCR stimulation. In striking concordance with our data, transient doubling of TfR surface expression or recycling at 5 min has been observed in adipocytes upon insulin stimulation [60], in macrophages upon macrophage colony-stimulating factor stimulation [61], in CHO cells after phorbol ester treatment [62], in malignant epithelial cells upon EGF stimulation [63] or in fibroblasts upon EGF or PDGF stimulation [64].

Thus, acute increase of iron importing capacity is a mechanism shared by many cell types in response to a large variety of stimulations, most probably to sustain cellular events taking place shortly after receptor engagement. An immediate consequence of upregulating TfR recycling or surface expression is the increase of the labile iron pool (LIP) [60, 65]. Similarly, in this study, we show that blocking transferrin import reduces free cellular iron. Interestingly, an increase in LIP is directly associated with production of reactive oxygen species (ROS) [37, 66]. Silencing or overexpression of the iron storage protein ferritin increases or reduces concomitantly the LIP and ROS [67, 68]. The fact that iron import can lead to production of ROS is in accordance with the slight but significant decrease in ROS that we measured in cell treated with anti-TfR antibodies.

Crucially, ROS are produced after the triggering of many surface receptors and represent a mechanism of signal transduction through their ability to inhibit phosphatases by oxidation of a cysteine residue of their active site [69, 70]. Thus, ROS could constitute the missing link between acute iron import by upregulation of transferrin recycling and early signalling. This would be consistent with the fact that TCR triggering leads to ROS generation within 15 min [71–73] and that TCR-induced ROS production is required for T cell activation [74]. In T cell too, ROS have been shown to inhibit phosphatases [75]. More importantly, inhibition of phosphatases by ROS downstream of TCR activation has been shown to promote Zap70 phosphorylation [73], in keeping with our observation that transferrin import is required for Zap70 phosphorylation 5 min after TCR triggering. Beyond T cells, inhibition of phosphatases by ROS has been shown to be required for integrin-mediated adhesion and cell spreading [76], in line with our results on LFA-1 adhesion to ICAM-1 coated surfaces.

In conclusion, our data suggest that early upregulation of iron import upon TCR triggering could sustain ROS production by transiently increasing the LIP. In turn, ROS would inhibit phosphatases and by doing so promote phosphorylation of Zap70 and PAK, eventually leading to increased LFA-1 mediated adhesion and more stable immunological synapses with antigen presenting cells. An interesting parallel can be established between this interpretation of our results and an ensemble of previous reports. Exactly like TCR triggering, stimulation by EGF or PDGF results in an acute upregulation of TfR recycling after 5 min [63, 64] and the signalling downstream of EGF and PDGF receptors relies on tyrosine phosphorylation through inhibition of phosphatases by ROS [70]. Thus, it appears that the upregulation of TfR recycling upon T cell activation is paralleled in multiple other cell types and signalling circumstances and might represent a widespread mechanism of regulating signalling via ROS production.

## Conclusions

In summary, our work shows that T cells upregulate their capacity to import iron immediately after triggering of the T cell receptor. They are doing so by transiently increasing surface expression and endocytic recycling of the receptor for iron transporter transferrin, through an endocytic pathway that involves the membrane proteins flotillins, Rab5- and Rab11-positive compartments and phosphatidylserine. Functionally, this fast and transient upregulation of iron import is correlated with formation of the immunological synapse through increased adhesion mediated by the integrin LFA-1 downstream of the kinases Zap70 and PAK. Our data further suggest that production of reactive oxygen species, through the labile iron pool could represent the link between import of iron and the activity of the kinases Zap70 and PAK.

## Methods

### Plasmids

Expression constructs encoding for human flotillin-1 or flotillin-2 were a gift from V. Niggli (University of Bern). TCRζ-PSCFP2 was provided by Prof. K. Gaus (University of New South Wales). PA-mCherry expression backbone was obtained from Clontech. TCRζ PA-mCherry was made as described in [15]. mCherry-Rab5 was a gift from Gia Voeltz (Addgene plasmid # 49,201). mCherry-Rab11a was a gift from Michael Davidson (Addgene plasmid # 55,124). pBGPa-CMV-GFP-OSBP PH domain was a gift from Tim Levine (Addgene plasmid # 58,724). LACT-C2 and 2xFYVE expression constructs were a gift from Prof. Rob Parton (University of Queensland).

### Cell culture

Jurkat T cells (Clone E6.1) and flotillin-1 and flotillin-2 knock-out Jurkat cell lines were cultured in RPMI 1640 medium (Pan Biotech) supplemented with 10% (vol/vol) FCS (Gibco). Flotillin-1 and flotillin-2 knock-out Jurkat cell lines were generated as described in [15]. Cells were transfected with 1 μg DNA per 200,000 cells, 18–20 h prior to imaging using the Neon electroporation kit (Invitrogen).

Before imaging, cells were incubated for 10 min at 37 °C on 18-mm glass-coated surfaces (Marienfeld, #0,117,580) that were prepared by incubating with poly-L-lysine (Sigma, #P8920) for 30 min at room temperature, then 10 μg/ml anti-CD3ε (eBioscience, #16–0037) and anti-CD28 (eBioscience, #16–0289) antibodies for T cell activation. For live cell imaging, cells were imaged from 10 to 40 min after their deposition on the coverslips.

For imaging transferrin internalisation, transfected Jurkat T cells were activated for 5 min, transferred onto ice, media exchanged for fresh cold media with 25 μg/ml transferrin-Alexa488 (Jackson ImmunoResearch, #009–540-050) or transferrin-Alexa647 (Jackson ImmunoResearch, #009–600-050) added, incubated for 5 min, media exchanged twice with fresh cold media, then transferred to 37 °C and imaged.

### Antibodies

Anti-CD3ε (clone OKT-3; eBioscience, #16–0037), anti-CD28 (clone CD28.2, eBioscience, #16–0289), anti-pPAK1/2 (1:1000, Cell Signaling Technologies (CST #2601 T)), anti-pZap70 (1:1000, CST #2701P), anti-pLAT (1:1000, CST#3584S), anti-pSrc family (1:1000, CST#2101), anti-pSLP-76 (1:1000, Abcam (#ab75829)), anti-pVav1 (1:1000, #ab47282), anti-CD11/CD18-FITC (1 μg/ml, #ab13219), anti-beta-actin (1:5000, #ab6276), anti-CD69-PE (1:100, Serotec), anti-CD25-APC (1:100, Biolegend, #302,610), anti-TfR-biotin (clone OKT-9; eBioscience, #13–0719-82), anti-TfR (clone M-A712, 10 μg/ml, BD Biosciences #555,534), anti-CD18-PE (1:50, BD Biosciences #555,924) and anti-CD29-PE (1:75, clone HUTS-21; BD Biosciences, #556,049).

### Preparation of primary human cells

Blood donation for research purposes was approved by the local ethics committee and individual donors gave written consent. PBMCs were enriched using the Vacutainer CPT system (BD Biosciences, #362,782). Then, T cells were enriched by negative magnetic sorting using the Pan T cell Isolation Kit (Miltenyi Biotec, #130–096-535). Pan T cells were used for experiments directly after isolation or cultured in RPMI, supplemented with 1% Penicillin/Streptomycin and 5% FCS if not indicated differently. For experiments involving expanded primary T cells, 1 × 10^6^ isolated T cells per ml were incubated with anti-CD3/anti-CD28-coated Dynabeads (Invitrogen, #11161D) in 1:1 ratio and 30 U/ml IL-2 were added. After 3 days, Dynabeads were magnetically removed and 1 × 10^6^ cells per ml were cultured for 4 more days with 30 U/ml IL-2.

### Microscopy

Fixed and live-cell confocal microscopy were performed on a Leica SP5 (Leica Microsystems) or Zeiss LSM780 laser-scanning confocal microscope (Zeiss) that are equipped with an argon laser (405, 488 nm), a diode pump solid state laser (561, 647 nm) and a live-cell incubation chamber (Pecon). GFP constructs and Alexa488-labelled proteins were excited using the 488-nm line of the argon laser source, while PA-mCherry- and mCherry-tagged proteins were excited with the 561-nm laser line. Images were acquired with a 100 × 1.4NA DIC M27 Apo-Plan oil immersion objective (Zeiss) and GaAsP-PMTs in simultaneous, bidirectional scanning mode. For each channel, the pinhole was set to 1 Airy Unit. Confocal photoactivation was achieved by illuminating a region of the cell outer membrane with a 7.2 μW 405-nm laser pulse with 12.24 μs pixel dwell time.

Live-cell TIRF images for vesicle (co-)fusion analysis were acquired on an ELYRA total internal reflection fluorescence microscope (Zeiss) with a 100 × oil-immersion objective with a numerical aperture of 1.46. Images were captured with a 20-ms exposure time.

Live-cell TIRF images for quantification of transferrin in flotillin-compartment were acquired on a DMi8 (Leica Microsystems) equipped with an Infinity TIRF module, a 405-nm diode laser, a 488-nm solid state laser, a 561-nm diode pumped solid state laser and a 638-nm solid state laser. Fast two-channel live cell imaging was performed using W-VIEW GEMINI image splitting optics with filters for spectral separation of GFP and mCherry (Hamamatsu, # A12801-01). Images were acquired with a HC PL APO 100 × /1.47 oil-immersion objective (Leica Microsystems) and fluorescence was detected with a DFC9000GTC sCMOS camera (Leica Microsystems). GFP was excited with the 488-nm laser; transferrin-Alexa647 was excited with the 638-nm laser. TIRF angle was adjusted to 100-nm penetration depth for 488-nm excitation light. Signals from GFP and transferrin-Alexa647 were separated with the W-VIEW GEMINI image splitting optics and simultaneously recorded on one half of the camera chip.

### Image analysis

Vesicle count and cross channel nearest neighbour distances for confocal experiments were determined with a custom Matlab vesicle tracking and cross-channel nearest-neighbour distance evaluation software as detailed in [15]. A GUI application and all source code for this analysis is freely available from https://github.com/PRNicovich/PAVesT.git.

Vesicle co-fusion events were visually identified and counted as containing transferrin-Alexa488 if a decrease in mean maximum intensity from the last three frames was greater than 10% compared to the maximum intensity of the initial frame as quantified by the analysed particles measure in FIJI.

Mean dispersion was calculated using a custom FIJI plugin, which evaluates endosome dispersion by calculating the intensity weighted measure of the average pixel distance from the centre of mass of the cell.

Transferrin entry into Rab5/11 endosomal compartments and transferrin content in flotillin-positive compartment was quantified in FIJI by creating a thresholded mask of endosomal compartments using the Rab or flotillin channel, respectively. The background set to zero, then the transferrin channel divided by the Rab/flotillin mask to calculate the mean transferrin intensity present in the desired compartments.

### Treatment for transferrin-binding inhibition

Jurkat T cells and expanded primary T cells were treated for 16 h prior to the start of experiments involving transferrin-binding inhibition. Treatment was continued during the experiments. First, cells were transferred into reduced-serum medium (Jurkat: 2% FCS, primary T cells: 5% FCS) to lower the amount of available iron-loaded transferrin. Additionally, in order to block the transferrin receptor (TfR, CD71) and inhibit binding of iron-loaded transferrin, cells were treated with 10 μg/ml anti-TfR (clone M-A712).

### Transferrin uptake assay

Jurkat T cells were collected and re-suspended in serum-free RPMI containing 30 μg/ml transferrin-Alexa647 (Jackson ImmunoResearch, #009–600-050) and supplemented (activated) or not (resting) with 1.5 μg/ml anti-CD3ε (OKT-3) + 1 μg/ml anti-CD28 (CD28.2). Cells were then incubated for 0 min or 30 min at 37 °C, cooled down on ice and washed twice with cold FACS buffer (PBS + 2% FCS) to remove unbound transferrin-Alexa647. Subsequently, fluorescence of at least 1 × 10^4^ cells per sample was analysed at a LSR II flow cytometer (BD Biosciences). Samples were kept on ice at all times before measuring. SYTOX Blue (0.1 μM; Invitrogen, #S11348) was added directly before measuring for identification of dead cells. The ratio of MedianFI of activated/resting cells was calculated for 0 min and 30 min timepoints.

To assess efficacy of anti-TfR treatment, Jurkat and primary T cells were treated or not with 10 μg/ml anti-TfR (M-A712) and activated for 20 h on plate-bound anti-CD3ε (OKT-3) and anti-CD28 (CD28.2). Transferrin-Alexa647 (30 μg/ml) was added for the last 90 min of incubation. Subsequently, activated cells were re-suspended, washed twice with PBS and at least 1 × 10^4^ cells per sample were analysed using a LSR II flow cytometer (BD Biosciences).

### Western blot

Jurkat T cells were cultured overnight in RPMI + 2% FCS supplemented or not with 10 μg/ml anti-TfR (M-A712). Next, Jurkat T cells were collected and re-suspended to 1.5 × 10^6^ cells per sample (200 μl) using the corresponding supernatant (ctrl versus anti-TfR). Similarly, primary T cells were pre-treated (RPMI + 5% FCS ± anti-TfR), collected and re-suspended to 4 × 10^6^ cells per sample (200 μl). Activating antibodies (Jurkat: 1.5 μg/ml anti-CD3ε (OKT-3) + 1 μg/ml anti-CD28 (CD28.2); primary T cells: 5ug/ml anti CD3ε (OKT-3) + 5 μg/ml anti-CD28 (CD28.2)) were added to the samples on ice. For activation, samples were incubated at 37 °C in a thermomixer with shaking (300 rpm) for the indicated times. Subsequently, pre-heated 5 × sample buffer (225 mM Tris–HCl pH 6.8, 5% SDS, 50% glycerol, 0.05% bromophenol blue, 4% β-ME) was added (50 μl) to lyse the cells for 10 min at 95 °C. Samples were frozen and thawed once. SDS-PAGE was followed by blotting using the Trans-Blot® Turbo™ Transfer System (BioRad) with 1A/25 V for 30 min onto Nitrocellulose Blotting Membranes (Amersham Protran 0.45 μm, GE Healthcare, #GE10600002). After washing with PBS-T (PBS + 0.02% Tween20), membranes were blocked for 1 h with ROTI-Block solution (Carl Roth, #A151.2). Depending on the target proteins, membranes were cut and subsequently incubated with primary antibody diluted in PBS with 0.05% Tween20 and 3% BSA overnight at 4 °C. Next, membranes were washed 3 times with PBS-T and then incubated for 1 h at RT with the corresponding secondary HRP-conjugated antibody diluted in PBS-T with 5% milk powder. Finally, membrane (-parts) were washed 3 times with PBS-T and then developed using Clarity Western ECL Substrate (Bio-Rad, #1,705,060) or SuperSignal West Femto Substrate (Thermo Scientific, #34,096). Band intensities were quantified relative to the brightest band on the blot and normalised to corresponding beta actin band intensity. Raw images of all used developed membranes are shown in Additional file 2, uncropped blots.

### CD69-CD25 up-regulation

Jurkat cells were seeded at 4 × 10^5^ per ml in RPMI with 2% FCS. Primary human T cells were seeded at 1 × 10^6^ cells per ml in RPMI with 1% Penicillin/Streptomycin and 5% FCS. Before activation cells were left untreated or treated for 24 h with 10 μg/ml anti-TfR (M-A712). To activate Jurkat or primary human cells, they were seeded in flat bottom 96 wells previously coated for 1 h with anti-CD3ε (10 μg/ml; clone OKT-3) and anti-CD28 (10 μg/ml; clone CD28.2). Non-activated (resting) controls were seeded in uncoated wells. After a defined time of activation or incubation in resting conditions, cells were re-suspended, washed once with PBS and stained for 20 min at 4 °C with anti-CD69-PE (1:100), anti-CD25-APC (1:100) or a combination thereof, diluted in FACS-buffer (PBS, 2% FCS). Samples were washed and at least 3 × 10^3^ cells were measured on a LSR II flow cytometer (BD Biosciences) equipped with violet (405 nm), blue (488 nm) and red (633 nm) lasers. SYTOX Blue (Invitrogen, # S34857) was added directly before measuring for identification of dead cells. Lymphocytes were gated based on scatter signals. Then, live cells were selected in a FSC/SYTOX blue dot-plot and median fluorescence intensities of PE and APC channels were determined. For each condition, the MedianFI of the corresponding unstained sample was subtracted from the mean of technical replicates before analysis.

To assess upregulation of CD25 after 96 h, corresponding “resting” cells were stained for surface CD25 and measured prior to activation after 24 h-treatment with anti-TfR. “Activated” cells were measured after 96-h activation on anti-CD3ε (OKT-3)- and anti-CD28 (CD28.2)-coated 96 wells.

### TfR surface level detection

Jurkat cells were collected and re-suspended in cold RPMI + 2% FCS to a density of 2 × 10^6^ cells/ml. For “activated” condition, activating antibodies (1.5 μg/ml anti-CD3ε (OKT-3) and 1 μg/ml anti-CD28 (CD28.2)) were added to the cells in a twofold concentrated solution, resulting in a cell number of 1 × 10^6^ per ml during activation. Plain RPMI + 2% FCS was added in the “resting” samples to yield 1 × 10^6^ cells per ml final concentration. In experiments with expanded primary T cells (d7), activation was performed with using 5 μg/ml and anti-CD3ε (OKT-3) and anti-CD28 (CD28.2) final concentration. Activation of cells was allowed for defined durations (5–60 min) by transferring them to 37 °C. Corresponding “resting” cells were incubated at 37 °C as well. Zero-minute timepoint samples were kept on ice. After the incubation at 37 °C, surface TfR was stained using different strategies depending on experimental setup. For experiments presented in Fig. 1, PFA (Polysciences, #18,814–20) was added to the samples to a final concentration of 3.7% and cells were fixed for 20 min at RT. Cells were washed twice and then surface TfR was stained with 2 μg/ml anti-TfR-biotin (OKT-9). Subsequently, samples were washed twice and stained with Streptavidin-Pacific Blue (4 μg/ml; Invitrogen, #S11222). Alternatively, (Fig. 3) cells were washed twice with cold serum-free RPMI and stained subsequently for 30 min in 30 μg/ml labelled transferrin (transferrin-Alexa647) diluted in serum-free RPMI. (Figs. 1 and 3) Before measuring at least 1 × 10^4^ cells with flow cytometry, cells were washed twice with PBS and re-suspended in FACS buffer.

### TfR recycling

Jurkat T cells or expanded primary T cells (d7) were collected, re-suspended in RPMI + 2% FCS to a density of 2 × 10^6^ cells/ml and incubated for 90 min with biotinylated anti-TfR (2 μg/ml, OKT-9) at 37 °C, to allow uptake of biotinylated anti-TfR (feeding). Subsequent steps, except recycling phase, were performed on ice with cold solutions. After the “feeding” phase cells were collected, washed with RPMI + 2% FCS twice and subsequently incubated with unlabelled Streptavidin (4 μg/ml in PBS) to block surface exposed biotin-anti-TfR. Per treatment one sample was not treated with unlabelled streptavidin to assess surface levels of TfR at t = 0 min. After 15 min, Alexa-488-labelled biotin (1 μg/ml; Sigma, #30,574-1MG-F) was added to the samples to block all remaining accessible binding sites of streptavidin. Then, cells were washed twice and re-suspended in cold RPMI + 2% FCS (“resting”) or in RPMI + 2% FCS, supplemented with activating antibodies (1.5 μg/ml anti-CD3ε (OKT-3) and 1 μg/ml anti-CD28 (CD28.2)) (“activated”). Next, to enable recycling of antibody-labelled TfR, cells were incubated for 5–40 min at 37 °C, or kept on ice (0 min recycling). Cells were washed twice with cold PBS and re-suspended in cold PBS containing Pacific Blue-labelled Streptavidin (4 μg/ml; Invitrogen, # S11222). “Unstained” samples were re-suspended in cold PBS without labelled streptavidin. Staining was performed 20 min at 4 °C. After washing with cold PBS, the samples were resuspended in cold FACS buffer and at least 1 × 10^4^ cells were measured at a LSR II flow cytometer (BD Biosciences). Samples were kept on ice at all times before measuring. Prior to measuring, 0.1 μM TO-PRO-3 (Invitrogen, #T3605) was added for identification of dead cells. For Fig. 1B, the ratio of Pacific Blue MedianFI of activated/resting cells was calculated for all timepoints. To compare TfR recycling between WT and FlotKO cell lines upon activation, Pacific Blue MedianFI relative to t = 0 min was plotted in Fig. 3C.

### LIP measurement (Calcein-AM)

Jurkat T cells were incubated overnight in RPMI + 2% FCS supplemented or not with 10 μg/ml anti-TfR. Next, Jurkat T cells were collected, re-suspended to 1 × 10^7^ cells per ml and stained with 1 μM Calcein-AM (Invitrogen, C1430) for 20 min. Then, samples were washed twice with PBS and re-suspended to 1 × 10^6^ cells per ml in RPMI + 2% FCS supplemented or not with 10 μg/ml anti-TfR. Prior to measuring fluorescence intensity of at least 1 × 10^4^ cells at a LSR II flow cytometer (BD Biosciences) using the GFP filter set, 10 μg/ml of anti-CD3ε (OKT3) and anti-CD28 (CD28.2) were added for activation. Dead cells were excluded by addition of 0.1 μM TO-PRO-3 (Invitrogen, #T3605). Median fluorescence intensity of anti-TfR-treated samples was normalised to the intensity of the corresponding control-treated sample.

### ROS measurement

Jurkat T cells were incubated overnight in RPMI + 2% FCS supplemented or not with 10 μg/ml anti-TfR (M-A712). Next, Jurkat T cells were collected, re-suspended to 2 × 10^6^ cells per ml in the corresponding supernatant and stained with 5 μM CellROX green (Invitrogen, #C10444) for 30 min or left unstained. For activation, 1.5 μg/ml of anti-CD3ε (OKT3) and 1 μg/ml anti-CD28 (CD28.2) were added for the final 5 min of staining. Then, cells were fixed with 4% PFA (Polysciences, #18,814–20) for 15 min, washed twice with PBS and at least 1 × 10^4^ cells were analysed at a LSR II flow cytometer (BD Biosciences) using the GFP filter set.

### Adhesion assay on ICAM-I and VCAM-I

Jurkat cells were pre-treated with anti-TfR or left untreated as described above. For the experiment, 96 wells were coated with ICAM-I-Fc (2 μg/ml, diluted in PBS, R&D Biosystems, # 720-IC-050) or VCAM-I-Fc (4 μg/ml, diluted in PBS, Biolegend, #553,706) for 1 h at 37 °C. To determine the exact number of cells added per well, one well was left uncoated to recover all cells after the experiment (input). Coated wells were washed twice carefully with PBS. Subsequently, 1 × 10^5^ cells were added per well in 100 μl and incubated for 15 min to equilibrate temperature and pH. Then, activating mAb cocktail (anti-CD3ε + anti-CD28, 1.5 μg/ml and 1 μg/ml final, respectively), or plain medium or MnCl_2_ (1 mM final) was added into corresponding wells and incubated 30 min at 37 °C. Input samples were collected for counting directly after incubation. Next, remaining wells were carefully washed twice with medium and adherent cells were dissociated with enzyme-free dissociation buffer (Gibco, #13,151,014). Cells were then resuspended and counted using a LSR Fortessa flow cytometer (BD Biosciences).

### Conjugate formation

Raji B- and (control or anti-TfR treated) Jurkat T cells were stained with 5 μM CellTrace™ Violet (Invitrogen, #C34557 (1 × 10^6^ cells per ml)) or CFSE (Invitrogen, #C34554 (1 × 10^7^ cells/ml)) diluted in PBS for 20 min, respectively, with dye switching between experiments. Next, Raji B cells (1 × 10^6^/ml) were pulsed (+ SEE) or not (− SEE) for 30 min with 2 μg/ml Staphylococcal Enterotoxin E (SEE/Superantigen) (Toxin Technology, #ET404). Subsequently, cells were washed three times with RPMI + 2% FCS. For conjugate formation, stained Jurkat T cells and Raji B cells were mixed in a 1:1 ratio, pelletised for 4 min at 300 × g and incubated at 37 °C for 10 min. For analysis, pellets were carefully resuspended in 100 μl RPMI + 2% FCS, diluted in 200 μl PBS, and at least 1 × 10^4^ events were analysed using a LSR II flow cytometer (BD Biosciences). Because a 1:1 ratio of Jurkat T:Raji B cells was not exactly observed during flow cytometric analysis, the number of conjugates, defined as CTV + / CFSE + double-positive events, are displayed relative to the total cell number (single cells + conjugates) of the cell type which was in minority in the particular experiment. Unspecific conjugates, defined as % conjugates in samples with un-pulsed Raji B cells (− SEE), were subtracted from the corresponding + SEE values.

### Staining of integrins

To determine surface levels of LFA-I and VLA-4 in low- and high-affinity conformation, 1 × 10^5^ cells were distributed in round bottom 96 wells in 100 μl and incubated for 15 min to equilibrate temperature and pH. Then, directly labelled antibodies detecting CD18 (PE, Clone 6.7, 1:50 final) or high-affinity-LFA-I (FITC-mAb24 CD11/CD18, 1 μg/ml final) or high affinity CD29-PE (PE-HUTS-21, 1:75 final) were added together with plain assay medium or activating mAb cocktail anti-CD3ε (1.5 μg/ml) + anti-CD28 (1 μg/ml)) or MnCl_2_ (1 mM final) into corresponding wells and incubated for 10 min at 37 °C. Then, cells were rapidly cooled down on ice and pelletised in a pre-cooled centrifuge (5 min, 350 × g, 4 °C). Supernatant was discarded and cells were washed once with ice-cold PBS. After washing, cells were resuspended in FACS buffer and fluorescence of at least 1 × 10^4^ cells was analysed using a LSR Fortessa flow cytometer (BD Biosciences).

### Statistical analysis

All statistical analyses excepting time of divergence analysis were performed using GraphPad software (Prism v9). Statistical significance between datasets was determined by performing two-tailed, unpaired non-parametric Student’s *T*-tests, one-way ANOVA, two-way ANOVA or one-sample *t*-test. Graphs show mean values for either single cells or independent repeats as indicated, and error bars represent the SEM. In statistical analysis, *p* > 0.05 is not marked or indicated as not significant (n.s.), whereas statistically significant values are indicated by asterisks as follows: *p ≤ 0.05, **p < 0.01, ***p < 0.001, ****p < 0.001.

Time of divergence analysis was performed using a custom MatLab script. To determine the timepoint of divergence, pooled fluorescence intensity distributions of WT and FlotKO#1 were compared by means of the non-parametric Wilcoxon rank-sum test at each timepoint. Briefly, the null hypothesis that WT and FlotKO#1 data sets are from continuous distributions with equal medians is tested against the alternative hypothesis they are not. For each timepoint, a *p*-value is calculated, with the timepoint of divergence defined as the first timepoint after which all subsequent *p*-values are equal to or smaller than the considered significance level. *p ≤ 0.05, **p < 0.01.

## Supplementary Information

**Additional file 1:** **Supplementary figures 1-4.** **Figure S1.** Related toFig. 2. Exemplary depiction of transferrin-Alexa488 quantification in Rab5 andRab11a compartments. ** Figure S2. **Related to Fig. 4. TCR isincorporated into an endosomal network demarked by phosphatidylserine.  **FigureS3.** Related to Fig. 6 and Fig. 7. Kinetics of phosphorylation eventsdownstream of TCR in expanded primary T cells from individual donors.  **FigureS4.** Related to Fig. 7. Mobilisation ofintegrins for adhesion at the IS depends on functional iron uptake throughtransferrin-TfR axis.

**Additional file 2.** Uncroppedblots related to Figs 6&7.

## Data Availability

The datasets generated and analysed during the current study are available through the zenodo repository at https://doi.org/10.5281/zenodo.6113017.

## Abbreviations

CME: Clathrin-mediated endocytosis
IS: Immunological synapse
LIP: Labile iron pool
PFA: Paraformaldehyde
PI(3)P: Phosphatidylinositol 3-phosphate
PI(4)P: Phosphatidylinositol 4-phosphate
PS: Phosphatidylserine
ROS: Reactive oxygen species
SEE: Staphylococcal enterotoxin E
SEM: Standard error of the mean
TCR: T cell receptor
TCRζ: Zeta chain of TCR (CD274)
TfR: Transferrin receptor (CD71)
TIRF: Total internal reflection fluorescence microscopy
