# Supplementary material for: Rapid increase in transferrin receptor recycling promotes adhesion during T cell activation
Source: BMC Biol. 2022 Aug 24;20:189. doi: 10.1186/s12915-022-01386-0 (PMC9400314; doi:10.1186/s12915-022-01386-0)
Supplement: Supplementary file 1 — Additional file 1: Supplementary figures 1-4. Figure S1. Related toFig. 2. Exemplary depiction of transferrin-Alexa488 quantification in Rab5 andRab11a compartments. Figure S2. Related to Fig. 4. TCR isincorporated into an endosomal network demarked by phosphatidylserine. FigureS3. Related to Fig. 6 and Fig. 7. Kinetics of phosphorylation eventsdownstream of TCR in expanded primary T cells from individual donors. FigureS4. Related to Fig. 7. Mobilisation ofintegrins for adhesion at the IS depends on functional iron uptake throughtransferrin-TfR axis. [file 12915_2022_1386_MOESM1_ESM.docx]

# Supplementary information


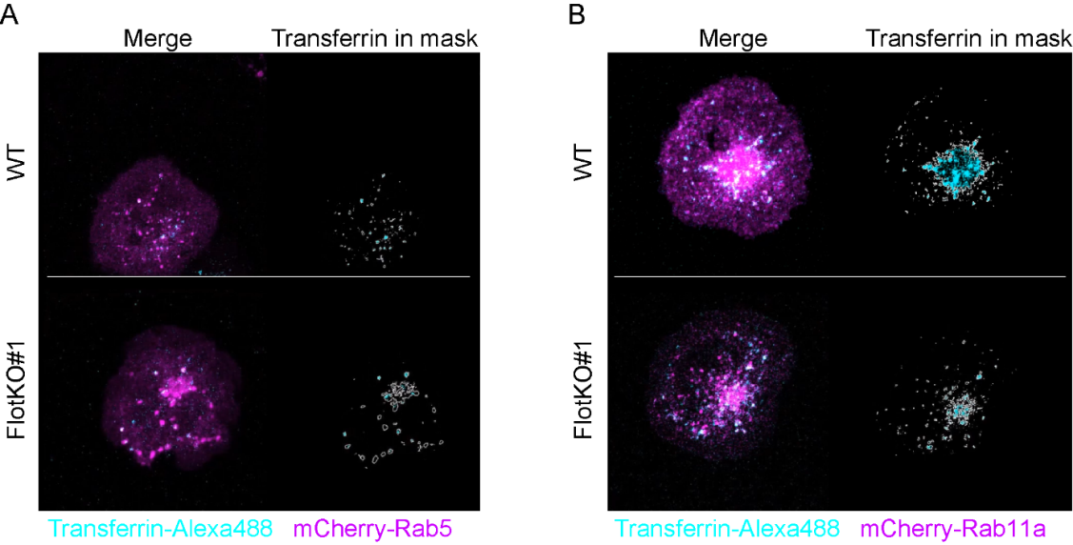
 **Figure S1.** Related to Fig. 2. Exemplary depiction of transferrin-Alexa488 quantification in Rab5 and Rab11a compartments. **A**-**B** WT or FlotKO Jurkat T cells expressing the indicated mCherry-tagged Rab proteins were incubated with transferrin-Alexa488 and imaged every 10 seconds for 60 frames. The mask (white outlines) was created from the mCherry-Rab5 signal (**A**) or mCherry-Rab11a signal (**B**), respectively.


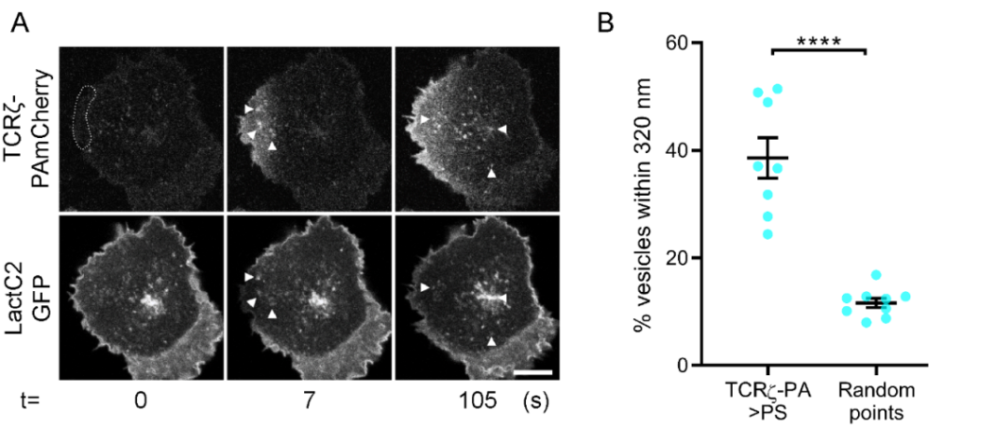
 **Figure S2.** Related to Fig. 4. TCR is incorporated into an endosomal network demarked by phosphatidylserine.
**A** TCRζ-PA-mCherry (top panel) and LactC2-GFP (bottom panel) before and after photoactivation in the representative confocal images dashed region. Arrows indicate co-occurring LactC2-GFP and TCRζ-PA-mCherry positive vesicles.
**B** Quantification of the percentage of photoactivated TCRζ-PA-mCherry vesicles within 320 nm of LactC2-GFP vesicles as determined by nearest neighbour analysis. Data points indicate individual cells from 2 independent experiments. Error bars indicate mean± SEM. ****= p<0.0001 from Student’s t-test.

**
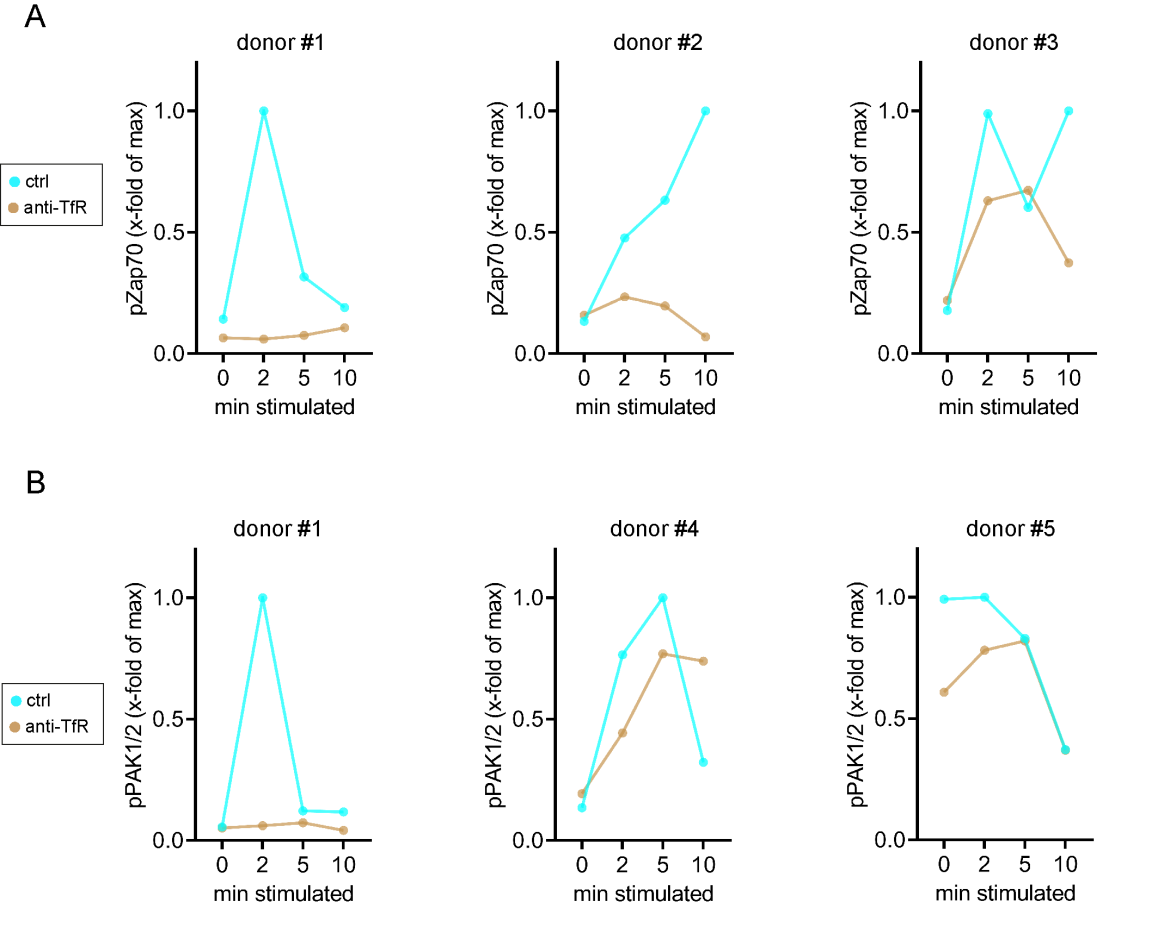

Figure S3.** Related to Fig. 6 and Fig. 7. Kinetics of phosphorylation events downstream of TCR in expanded primary T cells from individual donors. Primary T cell activation was accomplished with soluble anti-CD3ε + anti-CD28 for the indicated times before cell lysis and SDS PAGE. **A** After blotting, the nitrocellulose membrane was probed with anti-phospho-Zap70 (Y319). Beta actin was used as loading control. Depicted are the quantifications of pZap70 band intensities relative to corresponding beta-actin band intensities and normalised to the timepoint of highest Zap70 phosphorylation. **B** After blotting, the nitrocellulose membrane was probed with anti-phospho-PAK1/2 (T423/T402). Beta actin was used as loading control. Depicted are the quantifications of pPAK1/2 band intensities relative to corresponding beta-actin band intensities and normalised to the timepoint of highest PAK1/2 phosphorylation.

**
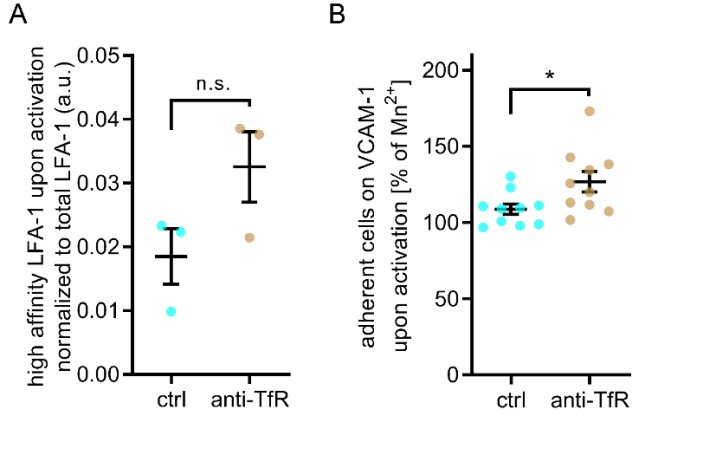
**
**Figure S4.** Related to Fig. 7. Mobilisation of integrins for adhesion at the IS depends on functional iron uptake through transferrin-TfR axis. **A** Quantification of high-affinity LFA-1 relative to total surface LFA-1, as measured by staining with conformation-sensitive antibody (clone mAb24), recognizing exclusively high-affinity LFA-1 and a non-conformation-sensitive antibody against CD18, to determine total LFA-1 surface levels. **B** Adherent anti-TfR or untreated Jurkat T cells on VCAM-I coating upon 30 min activation with soluble anti-CD3ε and anti-CD28 relative to the maximum adhesion capacity induced by addition of 1 mM MnCl_2_. Statistical significance determined with unpaired two-tailed Student’s t-test. * p<0.05;
n.s – not significant
