# Supplementary material for: Rapid increase in transferrin receptor recycling promotes adhesion during T cell activation
Source: BMC Biol. 2022 Aug 24;20:189. doi: 10.1186/s12915-022-01386-0 (PMC9400314; doi:10.1186/s12915-022-01386-0)

Jurkat pZap70

Replicate 1

aTfR | ctrl

Anti-pZap70

Anti-b-actin

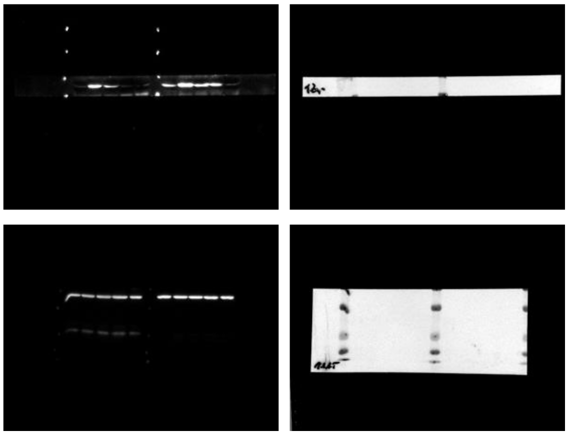

Replicate 2

aTfR | ctrl

Anti-pZap70

Anti-b-actin

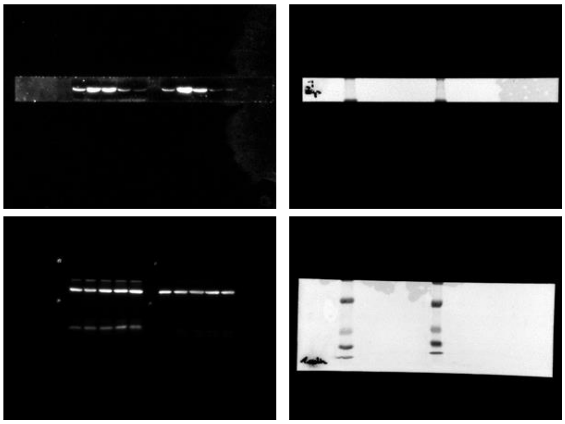

Replicate 3

aTfR | ctrl

Anti-pZap70

Anti-b-actin

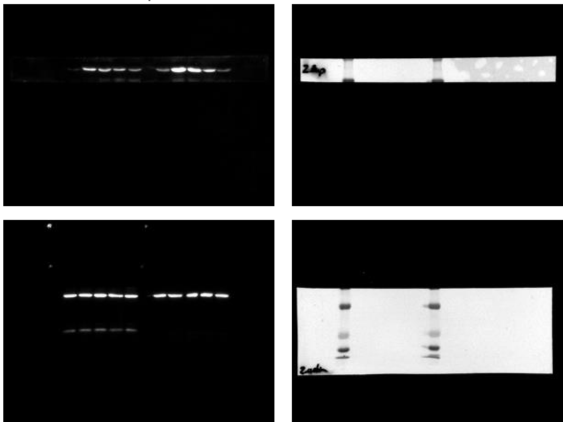

Replicate 4

aTfR | ctrl

Anti-pZap70

Anti-b-actin

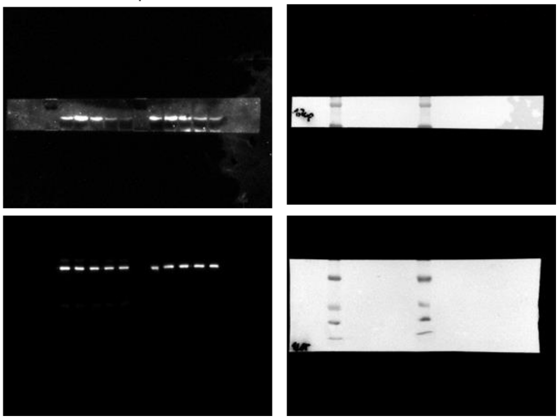

Replicate 5

aTfR | ctrl

Anti-pZap70

Anti-b-actin

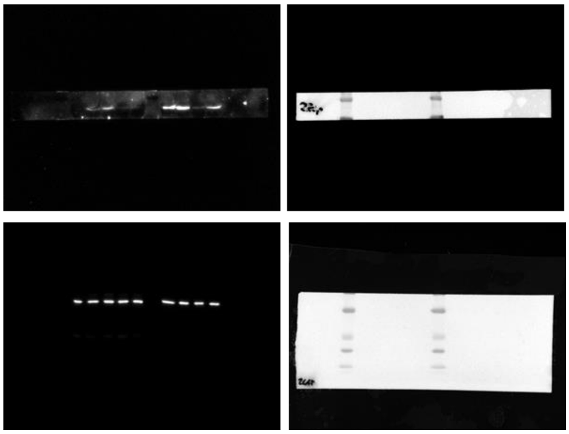

Jurkat pLat

Replicate 1

aTfR | ctrl

Anti-pLat

Anti-b-actin

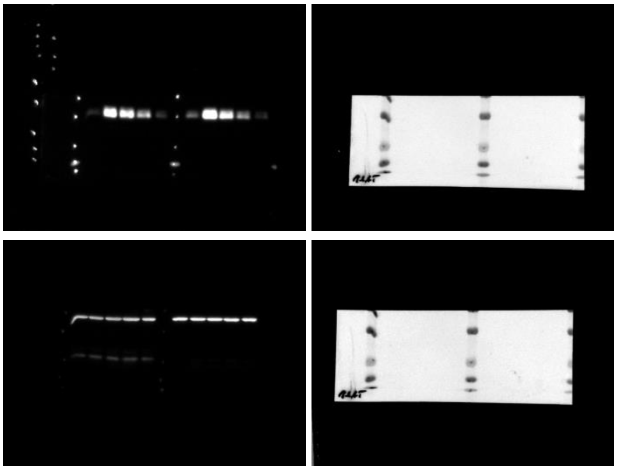

Replicate 2

aTfR | ctrl

Anti-pLat

Anti-b-actin

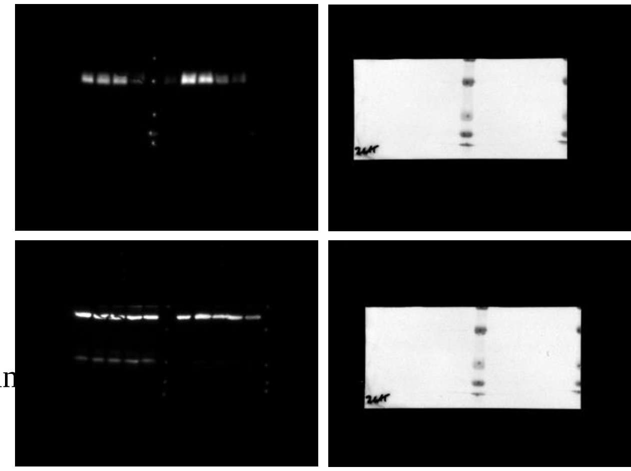

Replicate 3

aTfR | ctrl

Anti-pLat

Anti-b-actin

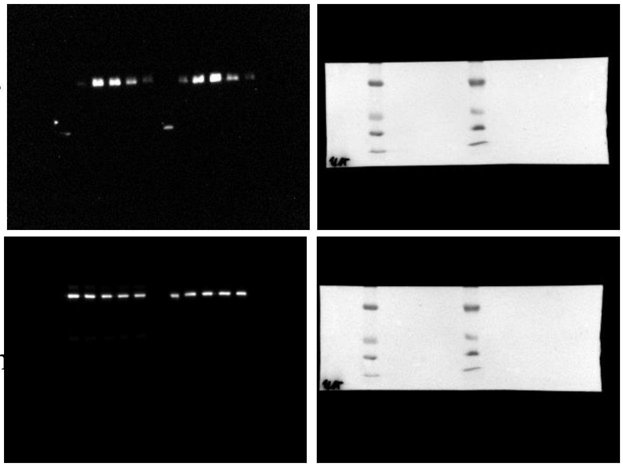

Replicate 4

aTfR | ctrl

Anti-pLat

Anti-b-actin

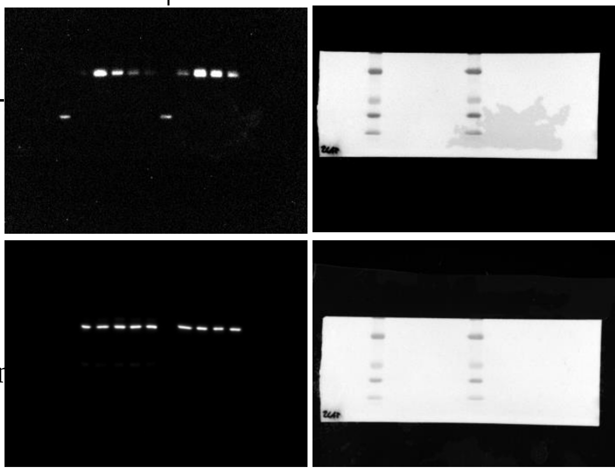

Jurkat pSLP76

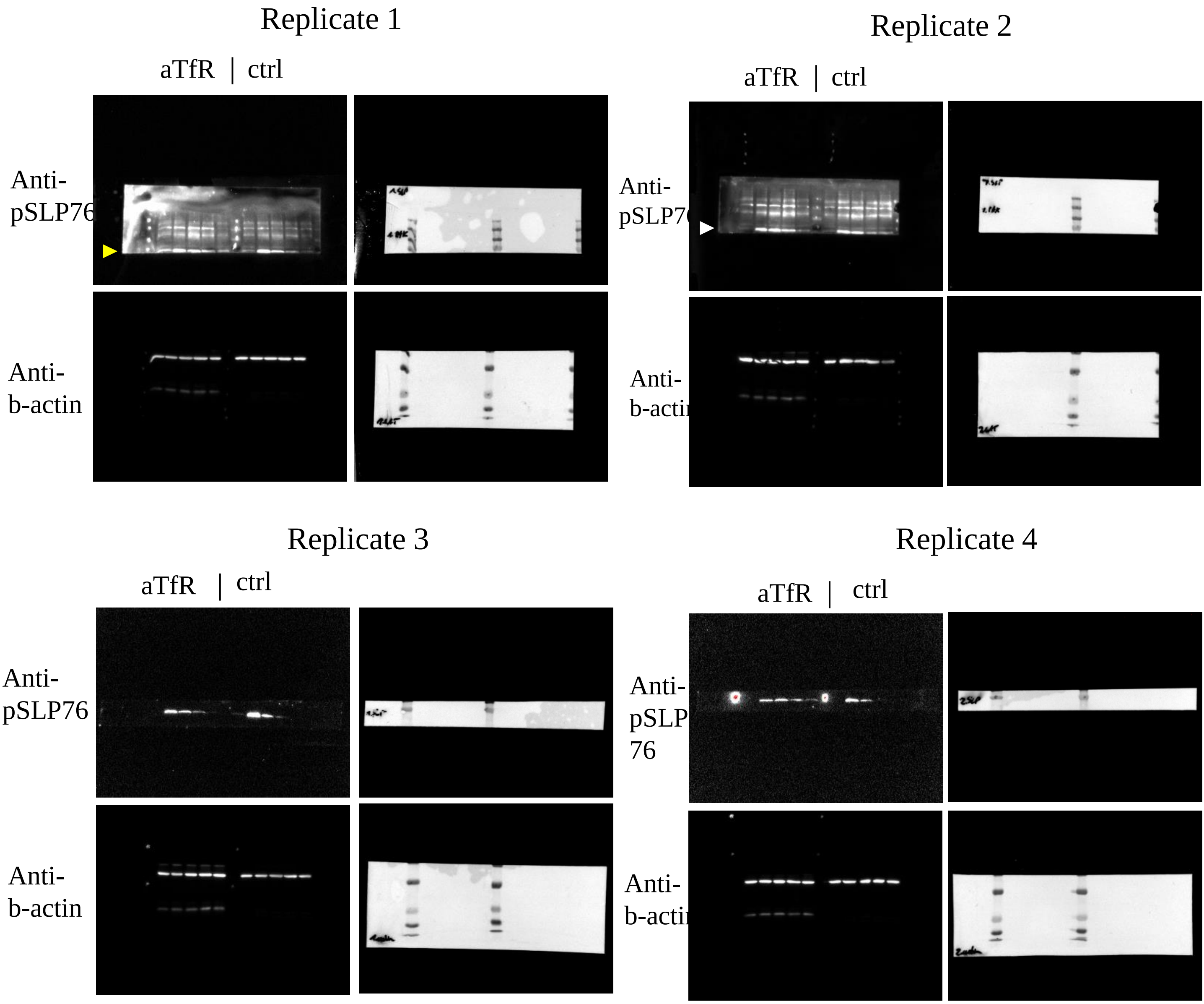

Jurkat pVav1

Replicate 1

aTfR | ctrl

Anti-pVav1

Anti-b-actin

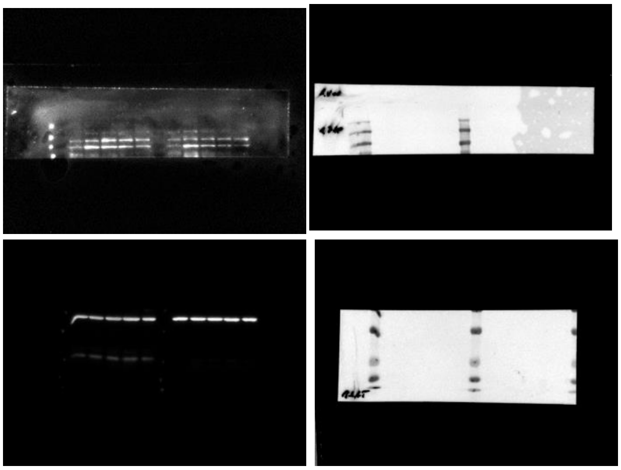

Replicate 2

aTfR | ctrl

Anti-Vav1

Anti-b-acti[illegible]

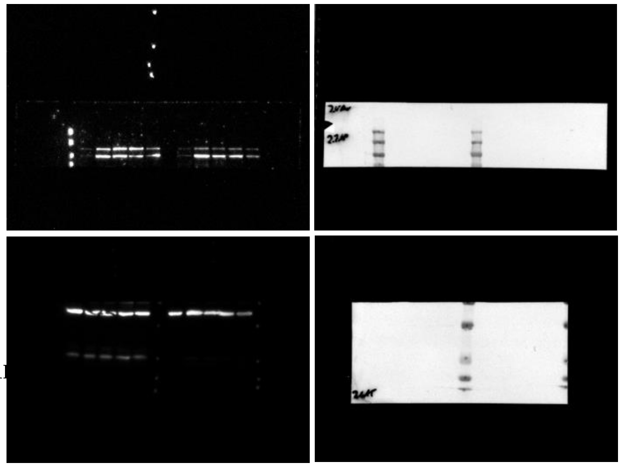

Replicate 3

aTfR | ctrl

Anti-pVav1

Anti-b-actin

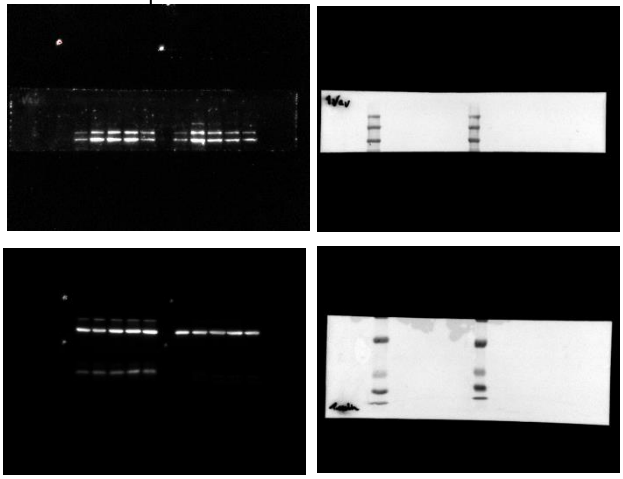

Replicate 4

aTfR | ctrl

Anti-pVav1

Anti-b-acti[illegible]

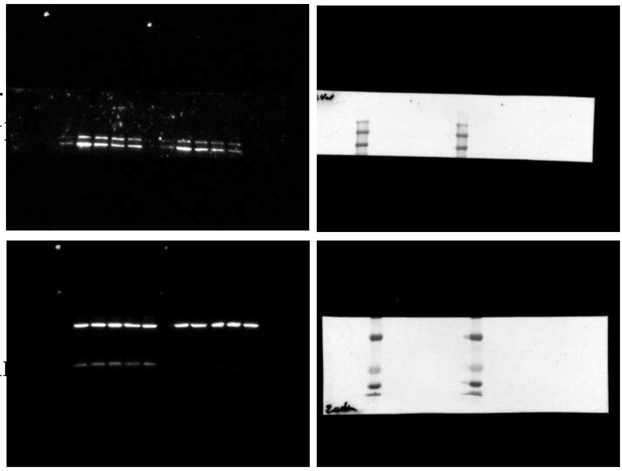

Jurkat pSrc family

Replicate 1

Ctrl | aTfR

Anti-pSrc fam

Anti-b-actin

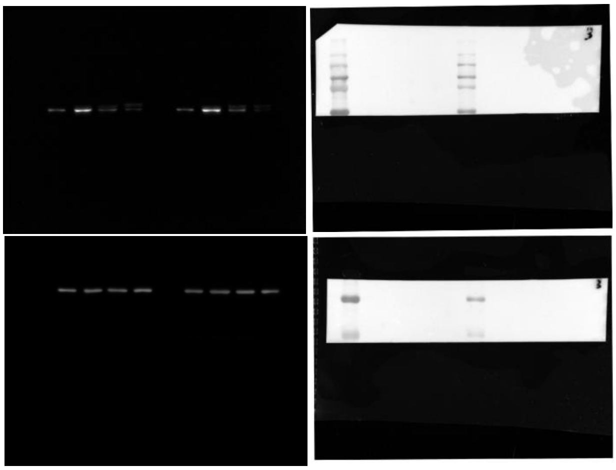

Replicate 2

Ctrl | aTfR

Anti-pSrc fam

Anti-b-acti

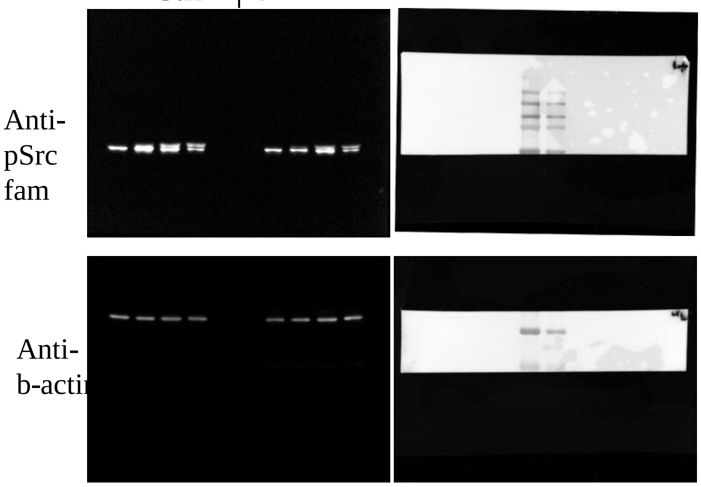

Replicate 3

Ctrl | aTfR

Anti-pSrc family

Anti-b-actin

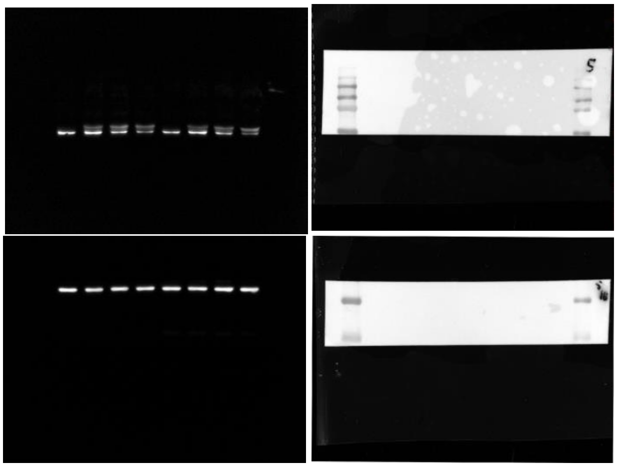

Primary cells pZap70

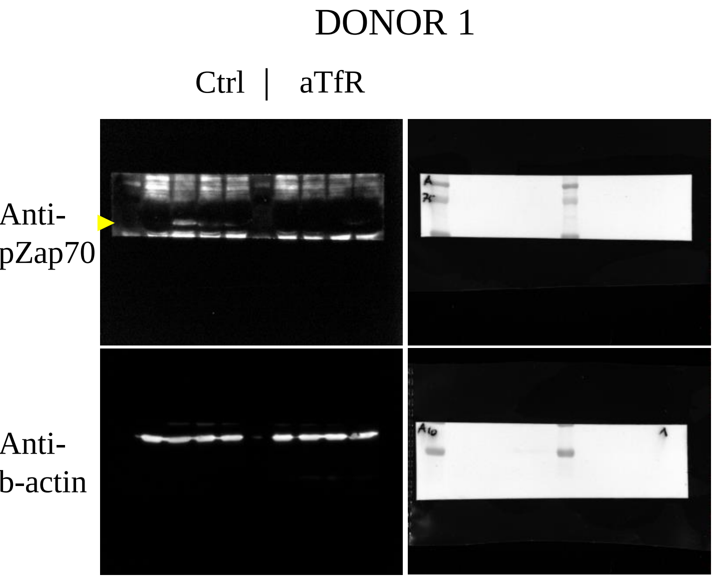

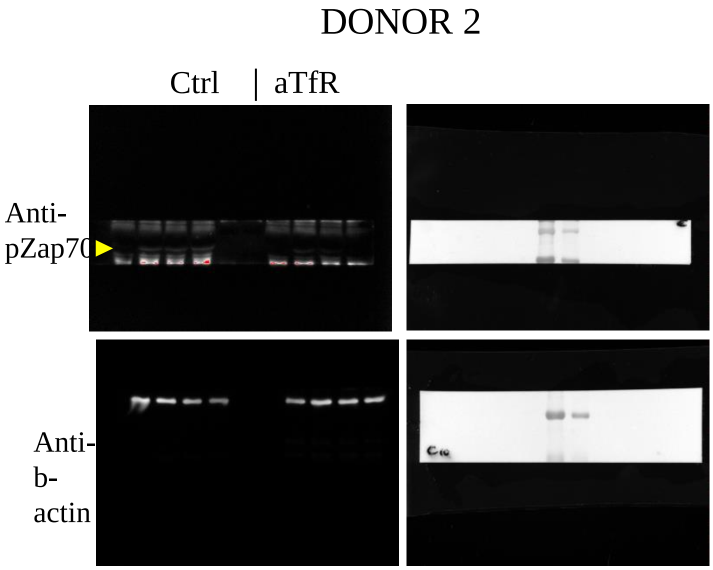

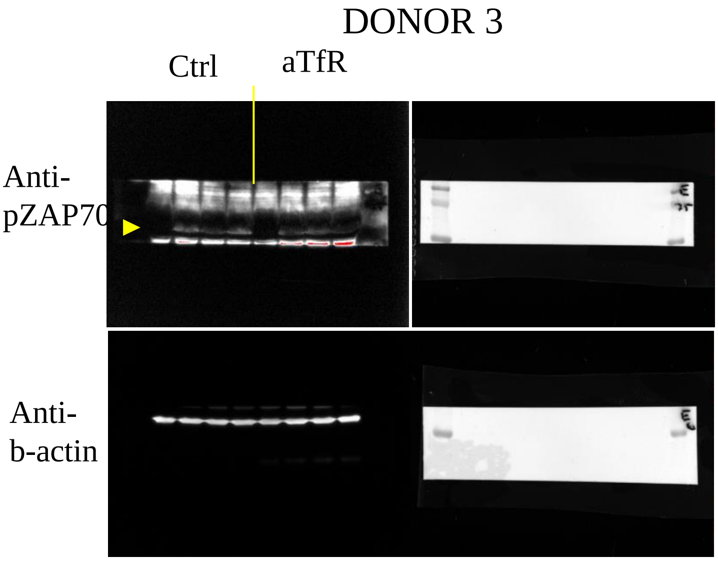

Jurkat pPAK1/2

Replicate 1

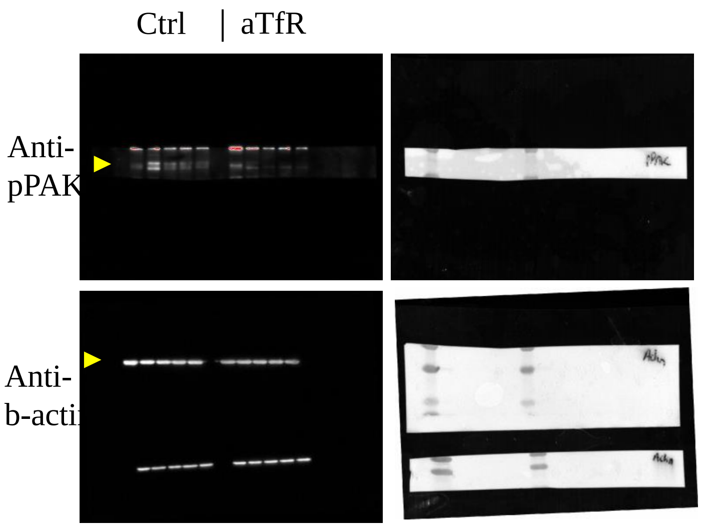

Replicates 2+ 3

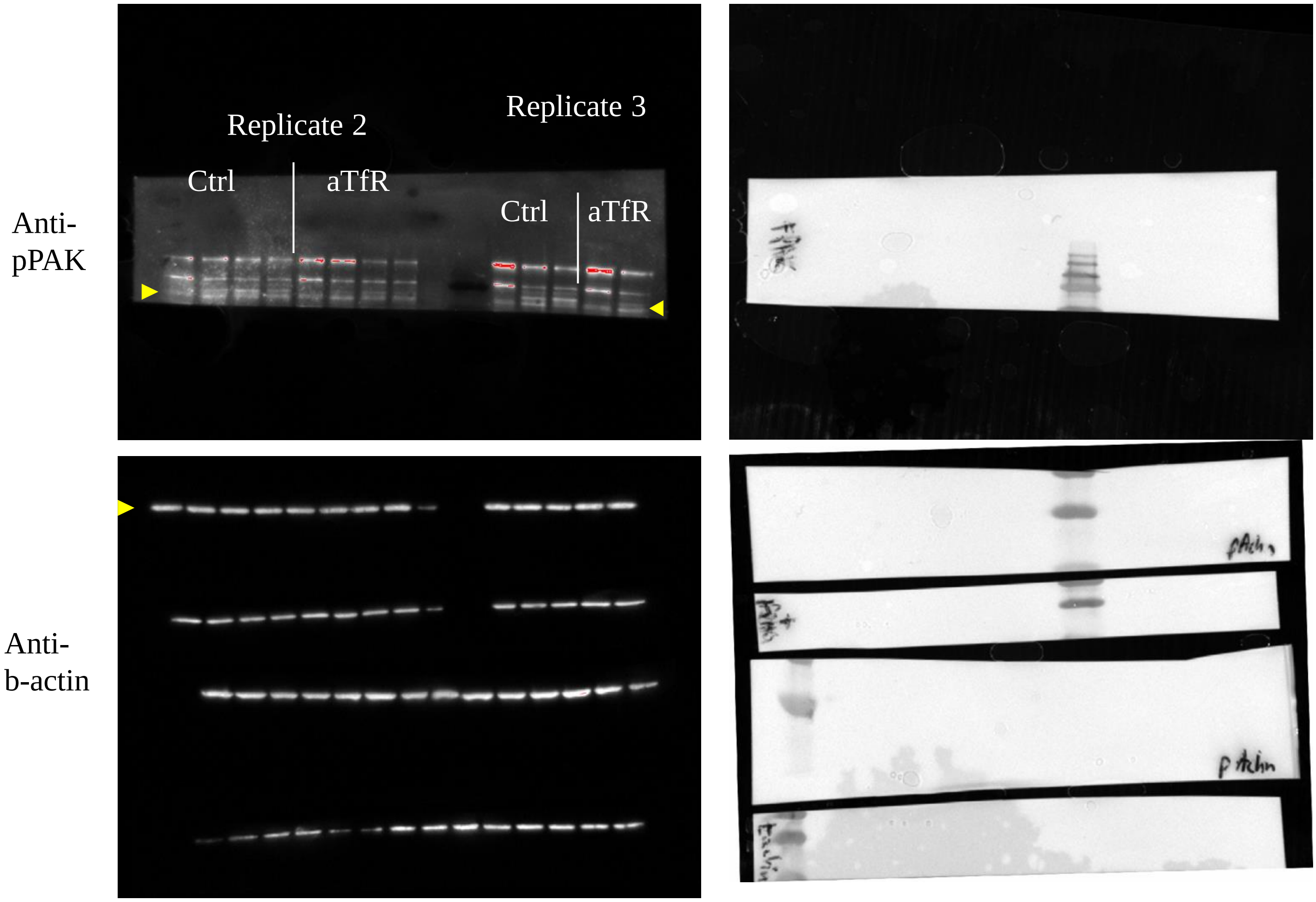

Primary cells pPAK

DONOR 1

Ctrl | aTfR

Anti-pPAK

Anti-b-actin

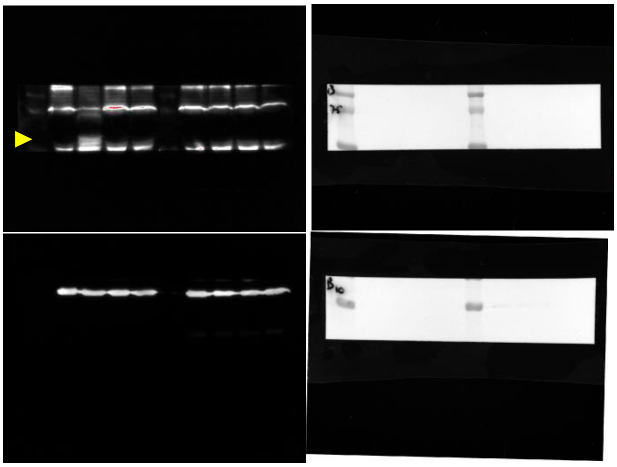

DONOR 4

Ctrl | aTfR

Anti-pPAK

Anti-b-actin

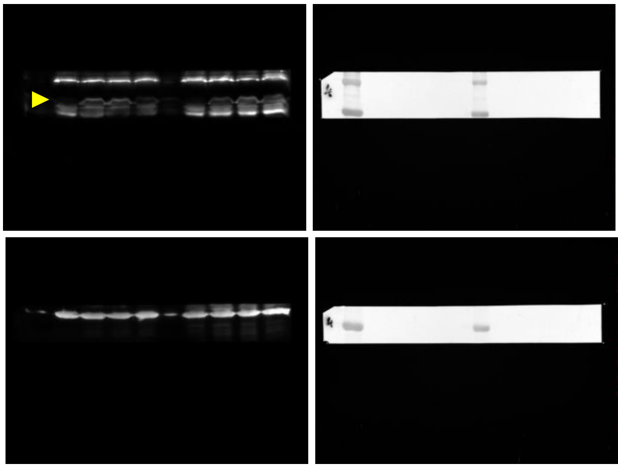

DONOR 5

Ctrl aTfR

Anti-pPAK

Anti-b-actin

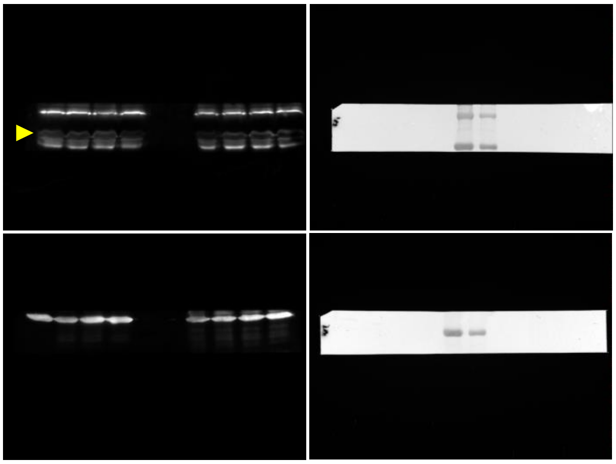

Supplement: Supplementary file 2 — Additional file 2. Uncroppedblots related to Figs 6&7. [file 12915_2022_1386_MOESM2_ESM.pdf]
